# PGC−1α Promoter Methylation in Parkinson’s Disease

**DOI:** 10.1371/journal.pone.0134087

**Published:** 2015-08-28

**Authors:** Xiaomin Su, Yaping Chu, Jeffrey H. Kordower, Bin Li, Hong Cao, Liang Huang, Maki Nishida, Lei Song, Difei Wang, Howard J. Federoff

**Affiliations:** 1 Department of Neuroscience, Georgetown University Medical Center, Washington DC, United States of America; 2 Department of Neurological Science, Rush University Medical Center, Chicago, Illinois, United States of America; 3 Innovation Center for Biomedical Informatics, Georgetown University Medical Center, Washington DC, United States of America; 4 Department of Neurology, Georgetown University Medical Center, Washington DC, United States of America; Johns Hopkins, UNITED STATES

## Abstract

The etiopathogenesis of sporadic Parkinson’s disease (PD) remains elusive although mitochondrial dysfunction has long been implicated. Recent evidence revealed reduced expression of peroxisome proliferator-activated receptor gamma coactivator−1 α (PGC−1α) and downstream regulated nuclear encoded respiratory complex genes in affected brain tissue from PD patients. We sought to determine whether epigenetic modification of the PGC−1α gene could account for diminished expression. In substantia nigra from PD patients but not control subjects, we show significant promoter-proximal non-canonical cytosine methylation of the PGC−1α gene but not an adjacent gene. As neuroinflammation is a prominent feature of PD and a mediator of epigenetic change, we evaluated whether the pro-inflammatory fatty acid, palmitate, would stimulate PGC−1α promoter methylation in different cell types from the CNS. Indeed, in mouse primary cortical neurons, microglia and astrocytes, palmitate causes PGC−1α gene promoter non-canonical cytosine methylation, reduced expression of the gene and reduced mitochondrial content. Moreover, intracerebroventricular (ICV) injection of palmitate to transgenic human α−synuclein mutant mice resulted in increased PGC−1α promoter methylation, decreased PGC−1α expression and reduced mitochondrial content in substantia nigra. Finally we provide evidence that dysregulation of ER stress and inflammatory signaling is associated with PGC−1α promoter methylation. Together, these data strengthen the connection between saturated fatty acids, neuroflammation, ER stress, epigenetic alteration and bioenergetic compromise in PD.

## Introduction

Despite intensive study, the etiology and pathogenesis of sporadic Parkinson’s disease (PD) remain obscure. A recent genome-wide meta-analysis demonstrated that there was consistent reduction in the expression of PGC−1α-dependent and ETC pathway genes in nigral tissues of PD patients [1]. The diminished expression of both PGC−1α transcript and gene product in PD patients has been independently corroborated [2]. PGC−1α is a transcriptional co-regulator with several functions including mitochondrial biogenesis in tissues such as liver, heart, adipose, brain, and kidney [3–5]. Recently PGC−1α has emerged as a link between mitochondrial dysfunction and transcriptional dysregulation in neurodegenerative diseases [6–8]. Mitochondrial dysfunction has long been associated with PD. Reduction of mitochondrial respiratory complex activities have been described in tissues from sporadic PD including brain [9, 10]. Ingestion or administration of the respiratory Complex I toxicant, MPTP, also results in loss of nigrostriatal dopamine neurons and parkinsonism. Given the extensive evidence for bioenergetic failure in PD, elucidating the mechanism(s) of PGC−1α pathway dysfunction in PD patients may contribute additional insights into the etiopathogenesis of disease.

Epigenetic mechanisms contribute in genetically complex non-Mendelian and also in monogenic Mendelian disorders, including PD, where discordance amongst monozygotic twins occurs. As such, epigenetic modifications provide a potential basis for environment and gene interaction. Nutrition, chemical exposures, and extrinsic physical factors affect disease risk through epigenetic mechanisms; these can occur during development or later in adult life when diseases like Parkinson’s present. This raises the possibility that epigenetic modifications of the PGC−1α promoter may account for down-regulation of gene expression in PD.

Free Fatty Acids can mediate epigenetic modifications, among other effects. Diets high in saturated FFAs alter DNA methylation in skeletal muscle [11]. Epidemiologic studies indicate that a diet high in saturated fat is a risk factor for PD [12, 13]. In mice fed a high–fat diet and challenged with a dopaminergic neurotoxicant, the magnitude of the nigrostriatal damage is exacerbated [14]. Diseases associated with systemic inflammation and elevated levels of plasma FFAs increase the risk for PD [15–18]. In head injury, an independent risk factor for PD [19], inflammation and increased FFAs are observed in the brain [20], with palmitic acid elevations approaching 0.2 mM [21]. Brain FFA uptake increases with aging [22], the greatest independent risk factor for sporadic PD, suggesting that metabolic alterations consequent to aging could be contributing factors.

Herein we test the hypothesis that PGC−1α promoter hypermethylation is an epigenetic contributor in sporadic PD. We demonstrate a significant increase of the promoter methylation in PD midbrain tissue that is accompanied by decreased PGC−1α gene and protein expression. Palmitic acid effectively promotes epigenetic modification of the PGC−1α gene in three primary CNS cell types (neurons, microglia and astrocytes). Additionally, ICV administration of palmitate to α−synuclein transgenic mice causes PGC−1α promoter hypermethylation in the substantia nigra (SN), decreased PGC−1α gene expression and reduction of mitochondrial content. Finally, we show that palmitate induces the unfolded protein response (UPR) and pro-inflammatory gene and protein expression in CNS glial cells, which may underlie FFA-mediated DNA methylation.

## Materials and Methods

### Human brain samples

Human brain frozen tissues were obtained from the NICHD brain and Tissue Bank for Development Disorders at the University of Maryland, Baltimore, MD and University of Miami. Georgetown University Institutional Review Board is acknowledged that the human brain samples are not from living individuals and any identifying information about the patients are not obtained. Georgetown University Institutional Review Board approval is not required. Fixed human brain tissues were provided by Department of Neurological Sciences at Rush University Medical Center. The Human Investigation Committee at Rush University Medical Center approved this study. All patients with Parkinson’s disease was diagnosed by movement disorder specialists at the respective institutions. Board-certified neuropathologists confirmed the clinical diagnosis. Age-matched control subjects were determined not to have clinical features of neurologic disease.

For tissue for immunocytochemistry, the brains were removed at autopsy and processed as described previously (Chu et al., 2006). Briefly, each brain was cut into 1 cm coronal slabs using a Plexiglas brain slice apparatus and then hemisected. The slabs were fixed in 4% paraformaldehyde for 5 days at 4°C. The left-side brain slabs were used for pathological diagnoses. The right-side brain slabs were cryoprotected in 0.1M PBS pH 7.4 containing 2% dimethyl sulphoxide, 10% glycerol for 48 h followed by 2% dimethyl sulphoxide and 20% glycerol in PBS for at least 2 days before sectioning. The fixed slabs were cut into 18 adjacent series of 40 μm thick sections on a freezing sliding microtome. All sections were collected and stored in a cryoprotectant solution before processing.

### Ethical statement

All animal housing and procedures were performed in compliance with guidelines established by the University Committee of Animal Resources at the Georgetown University. Georgetown University Institutional Animal Care and Use Committee (IACUC) have approved this study. Rodents were anesthetized with ketamine for all surgical procedures and collection of brain samples. After-surgical analgesia (carprofen) was administered to animals after implantation procedure. Prophylactic antibiotic treatment was initiated on the day of surgery. Animals were euthanized using CO_2_ followed by cervical dislocation.

Human brain section immunofluorescent labeling and optical density measurements

Five midbrain sections from each brain were incubated in the primary antibody Peroxisome proliferator-activated receptor gamma co-activator 1-α (PGC−1α; 1:500; AP08988PU-N; Acris Antibodies, Inc) overnight and the donkey anti-goat antibody coupled to DyLight 488 (1:200, Jackson ImmunoResearch) for 1 h at room temperature. The sections were mounted on gelatin-coated slides and allowed to air dry overnight. To block autofluorescence, the sections were rinsed in distilled water, dehydrated in 70% alcohol for 5 min, incubated in the autofluorescent eliminator reagent (2160; Millipore) for 5 min, and immersed in three changes of 70% alcohol. After rinsing in distilled water, the sections were cover slipped using polyvinyl alcohol with DABCO (Sigma-Aldrich).

Optical density measurements were performed according to our previously published procedures (Chu et al., 2009, 2012). All immunofluorescence labeled images were scanned with an Olympus Confocal Fluoroview microscope equipped with argon and transparent optics. With a 20 opticsasurements were performed according to our previously published procedures (Chu et al.,neuromelanin (NM), images were acquired at each sampling site in the substantia nigra pars compacta and were saved to a Fluoroview file. Following acquisition of an image, the stage moves to the next sampling site to ensure a completely non-redundant evaluation. Once all images were acquired, optical density measurements were performed on individual nigral neurons. To maintain consistency of the scanned image for each slide, the laser intensity, confocal aperture, photomultiplier voltage, offset, electronic gain, scan speed, image size, filter and zoom were set for the background level whereby autofluorescence was not visible with a control section. These settings were maintained throughout the entire experiment (Chu et al., 2012). The intensity mapping sliders ranged from 0 to 4095; 0 represented a maximum black image and 4095 represented a maximum bright image.

The PGC−1α immunoreactive somas with NM were identified, outlined, and measured using Fluoroview software. Five equi-spaced sections per case across the entire length of the substantia nigra were sampled and evaluated. Greater than 150 nigral cells were analyzed in per subject. To account for differences in background staining intensity, five background intensity measurements lacking immunofluorescent profiles were taken from each section. The mean of these five measurements constituted the background intensity that was then subtracted from the measured optical density of each individual neuron to provide a final optical density value.

### Animal study

The transgenic mouse model overexpressing human DM SYN overexpression under the control of the 9-kb rat tyrosine hydroxylase promoter was previously developed in our laboratory [23]. We have since induced homozygosity at the α-synuclein locus for this model (DMSYN). DMSYN mice were in C57BL6 background, male, at the age of 6 months old. The median weight was 30g (28–32 g). The animals were generated and bred in house. The treatment group included DMSYN mice with 0.5 and 1.0 uM palmitate respectively (n = 4/dose) and the control group were DMSYN mice with PBS treatment (n = 5). The sample size estimation was completed using the Newman-Keuls multiple comparison adjusted mixed effect model, with the proposed sample size of 4 mice per group, it provides at least 80% power to detect an effect size of 2.5 (about 30% mean difference) with Newman-Keuls multiple adjusted two-sided type I error = 5%. The animals were randomly allocated to experimental groups. The animals in the different experimental groups were treated and assessed by the order of control-treatment alternation.

Animals received osmotic mini pump (Alzet) implantation with a cannula aiming into the right lateral ventricle (AP:-0.4mm; ML: 1mm; DV: 2mm). One hundred microliters of solution (saline, 0.5 or 1 uM palmitate in saline) were consistently delivered to the brain at a flow rate of 0.25 ul/h for 14 days. Ketamine was used as analgesia during surgery. After surgery, animals were transported back to animal facilities. Animals were monitored daily for any advent events. All mice were sacrificed in the morning time on the same day under inhaled CO2 followed by cervical dislocation as per approved animal protocol. After the brain samples were taken from the animals, they were processed by a blinded technician. Brain samples were dissected and frozen in liquid nitrogen until RNA isolation procedure was performed. The experimental unit is a single animal. The primary experimental outcome was methylation status of the PGC−1α promoter. The secondary outcomes are PGC−1α gene expression and the ratio of mitochondrial DNA to nuclear DNA

The housing facility was specific pathogen free. Ventilated microisolator cages were used; the bedding material was corn cob. Three animals were housed per cage in a temperature-controlled (70°F ± 2°F) room wit a light/dark cycle (12hrs/12hrs; lights on at 7am). Acidified water and food Harland Teklad 2920 chow were provided *ad libitum*.

### Isolation and culture of primary mouse critical neurons, microglia and astrocyte

Primary cortical neuronal cultures were established from 16 days old embryonic C57BL6 mice. Dissociated cells were plated onto poly-D-lysine coated plates (6-well plates, 12×10^5^ cells/well) in Neurobasal medium supplemented with 2% B27, 50U/50 μg/mL penicillin-streptomycin and 2.0 mM Glutamax and grew at 37°C in 100% humidity, 95% room air/5% CO_2_. Every three days, half of the mediums were replaced by fresh ones.

To obtain primary microglia cultures, cerebral cortices of neonatal mice (1-day old; C57BL/6) were stripped of meninges and minced in hepes balanced salt solution (HBSS; Mediatech Inc., Herndon, VA). Cells were dissociated in minimum essential media (MEM; Invitrogen, Frederick, MD) containing Earle's salts, L-glutamine, 0.01% pyruvate, 0.6% glucose, 4% fetal bovine serum and 6% horse serum (complete medium), centrifuged, resuspended and plated into flasks containing 10 mL complete medium at a density of one brain per T75 flask. Cultures were grown at 37°C under 5% CO_2_. After 1 day, the flasks were tapped gently to remove cell debris, media removed and replaced with fresh complete media. Cultures were grown as above for approximately 12 days at which time the microglia were harvested by tapping the flasks and collecting the microglia-enriched containing medium. Microglia were pelleted by centrifugation (1000 rpm, 5 min), resuspended in MEM medium containing 0.01% pyruvate, 0.6% glucose, and 5% fetal bovine serum and enumerated. Primary microglial cells were plated in 6-well plates at a density of 1× 10^6^ cells per well. Immunocytochemical staining of the astrocyte enriched cultures with anti-Iba1 antibody indicated a purity of ≥ 98%.

To obtain primary cultures of astroglial cells, the mixed glial cultures, after the separation of microglia as described above, were detached with trypsin–EDTA and seeded in the same culture medium used for microglia. After at least five consecutive passages, cells were seeded (10^6^/well) into 6-well plates for experiments. Immunocytochemical staining of the astrocyte enriched cultures with anti-GFAP antibody indicated a purity of ≥ 98%.

### Total RNA, genomic DNA and protein extraction

Total RNA, genomic DNA and protein were extracted using All-in-One kit (Norgen Biotek, Thorold, Canada). Cell media were aspirated then the cells were washed in 2mL PBS. Cell lysates were prepared by adding 350 uL of lysis solution followed by 200 uL of ethanol. Up to 600 uL of the lysate were applied to the All-in-one spin column and spun at 14,000 g for 2 min. Flow through were retained and store at -20°C for protein purification. 400 uL of the RNA wash solution were added to the column and spin for 2 min. Total RNA were eluted with 50 uL of the RNA elution solution. Then 500 uL of the genomic DNA were added to the column and the columns were spun for 2 min. Genomic DNA were eluted with 100 uL of genomic DNA elution buffer adding to the column.

### Bisulfite Sequencing

The purified genomic DNA (200 ng) was treated with bisulfite according to the manufacturer's protocol (EpiTect Bisulfite Kit, Qiagen, Valencia, CA, USA). For amplification of the region from −361 to −90 of the PGC−1α promoter in mouse samples, the following primers were used: sense 5′ GGA TGG AAA ATA AAT TA AAA AAA AAA GAT TG 3′; antisense 5′ TCA CTC ACC CAA CCT CCC TTC TCC 3′. For Dhx15: sense 5′TTG GAG GTA AAA TAT AGT GTT TAG TG 3′; antisense 5′AAA CAA AAT CAC ACA ACT AAC AAA TAA C 3′; For amplification of the region from −358 to −60 of PGC−1α promoter in human samples, the following primers were used: sense 5′ TAT AGT TAT TTT GTT ATG AAA TAG GGA GTT TT G 3′; antisense 5′ CCA ATC ACA TAA CAA AAC TAT TAA AAA ATA A 3′. For GBA3: sense 5′ AAA TGG TTA AAA GTG GTT ATT TTT ATA GAG 3; antisense 5’ CAA AAC ACC CAT TTA CCT AAT ATT TTA C 3’. The resultant PCR fragments were purified from an agarose gel using MinElute Gel Extraction Kit (QIAGEN) and cloned into TOPO TA vector, according the manufacturer's protocol (Life Technologies). Individual clones were grown and plasmids purified using QIAprep Spin Miniprep Kit (QIAGEN). For each condition, 10–50 clones were sequenced using M13 Reverse promoter primer secured from the Genewiz service (Germantown, MD). Sequencing results were analyzed using MethTools V1.3 [24].

### Mitochondrial DNA content

The ratio of mitochondrial versus nuclear DNA was determined as described (Walker et al., 2005), with the following adaptations: briefly, 10 uL of purified DNA at 1 ng/uL was amplified in a 25 uLPCR reaction containing 1× SYBR Green Master Mix (Applied Biosystems) and 100 nM of each primer. The amplification was monitored in real-time using the ABI Prism 7000 Real-Time PCR machine (Applied Biosystems). The primers were designed to target nDNA (forward: CTT GCA GTG AGC CGA GAT T A; reverse: GAG ACG GAG TCT CGC TCT GTC) or mtDNA (forward: AAT ATT AAA CAC AAA CTA CCA CCT ACC T; reverse: TGG TTC TCA GGG TTT GTT ATA A).

### Microarray Gene Expression Analysis

17 microarray datasets of human brain samples were used for differential gene expression analysis in this study. They are publicly available in the Gene Expression Omnibus (GEO) under GEO accession numbers GSE6613, GSE7621, GSE8397 (two data sets), GSE20141, GSE20146, GSE20153, GSE20159, GSE20163, GSE20164, GSE20168, GSE20291, GSE20292, GSE20314, GSE20333, and GSE24378 [1]. Data analysis was performed using R and Bioconductor packages [25]. Each dataset was normalized separately using Robust Multi-array Average (RMA) implementation [26]. Two-sample t-test with Storey method for false discovery rate (FDR) was carried for statistical analysis. To determine if a gene is a differentially expressed, we used these criteria: (1) the fold change of PD/Normal, either up- or down-regulated, was at least 1.4-fold in order to filter out small changes in expression. (2) The difference between two groups was statistically significant when p and q value were at least less than 0.05 and 0.25, respectively.

### Western blot analysis

Cell lysate was homogenized in buffer containing 50mM HEPE (pH 7.6), 150 mM NaCl, 1% Tritox X-100, 1 mM Na3VO4, 10 mM NaF, 30 mM Na4P2O7, 10% (v/v) glycerol, 1 mM microcystin. Protein was determined by the BCA (bicinchoninc acid) protein assay kit from Piece (Thermo Fisher Scientific; Waltham, MA). Samples were resuspended in laemmli buffer, and proteins were separated on 12% SDS-PAGE. Proteins were transferred to polyvinylidenedifluoride membranes (Millipore; Billerica, MA) and suggest to western blot analysis. After incubation with primary antibody, membranes were washed and incubated with secondary antibody linked to horseradish peroxidase (Bio-Rad; Hercules, CA). Results were quantified by densitometry using ImageJ, 1.48V (NIH).

### ELISA analysis

TNFα in the culture media was quantified by ELISA following the manufacture’s instruction (Thermo Fisher Scientific, Waltham, MA). Briefly, 50 uL of standards or samples was added to each well in duplicate of 96-well plate. Then 50 uL of biotinylated antibody reagent was added to each well. The plate was incubated at room temperature for 2 hours followed by wash for five times. 100 uL of dilute streptavidin-HRP was added to each well. The plate was incubated at room temperature for 30 min followed by wash for five times. Then 100 uL of TMB substrate was added to each well. The plate was developed in the dark at room temperature for 30 minutes. 100 uL of stop solution was added to the well to stop the reaction. The absorbance was measured on a plate reader.

### Statistics

Quantitative data are presented as the mean ± SEM. Statistical significance was either assessed via an unpaired Student’s t test or an ANOVA test with Student-Newman-Keuls post hoc analysis. Assessments were considered significant with a p < 0.05.

## Results

### Hypermethylation of the PGC−1α promoter in sporadic PD substantia nigra

**Fig 1:**
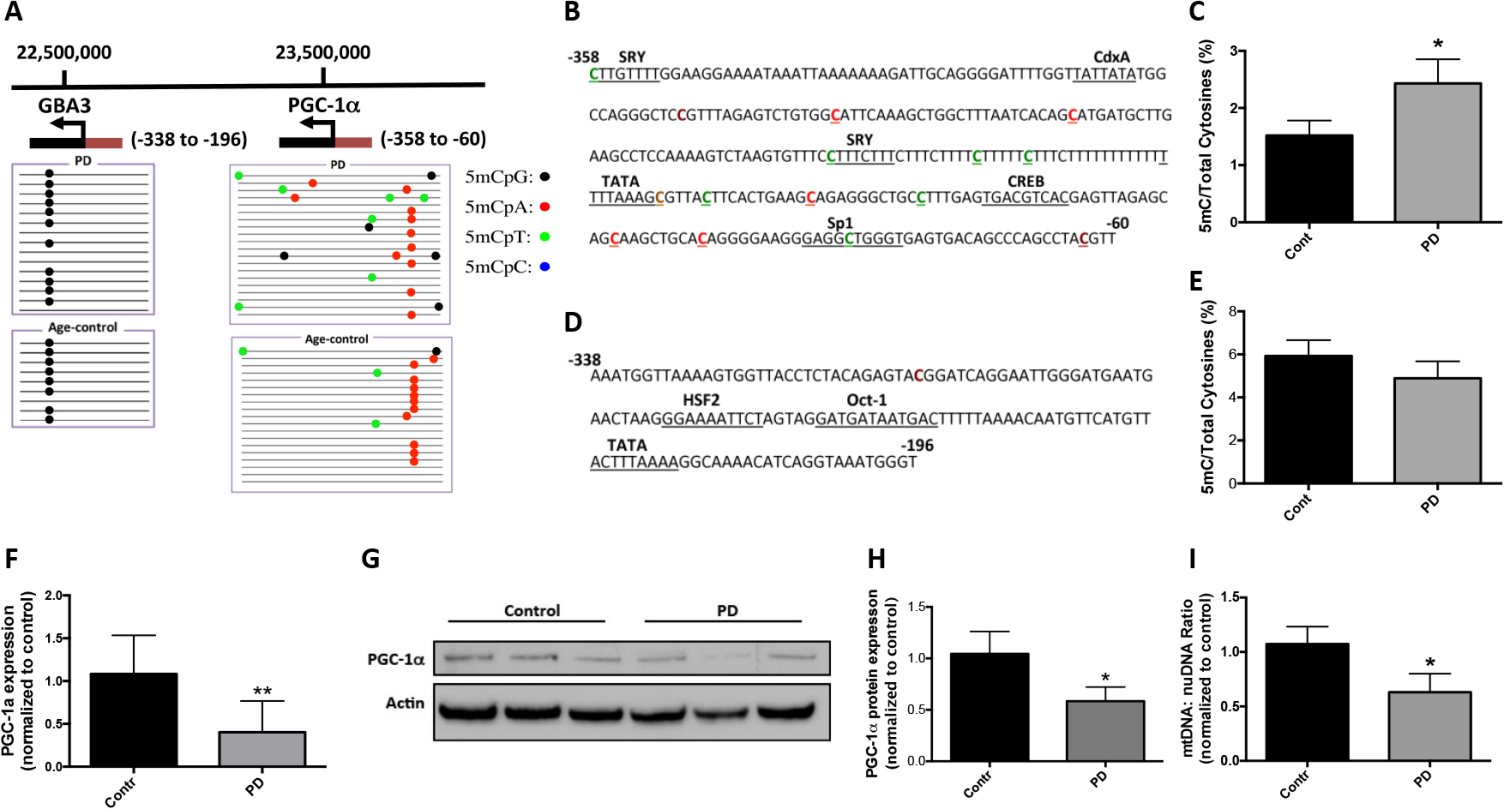
Methylation analysis of the PGC−1α promoter in PD patients and age-matched controls. **A**. Graphical presentation of the genomic localization of the PGC−1α and *GBA3* promoters on chromosome 4. The transcription start site (arrow), transcription units (black box) and sequenced promoters (red box) are shown for both genes. *GBA3* is an adjacent to the PGC−1α gene. Genomic DNA was isolated from substantia nigra from PD and age-matched controls for bisulfite sequencing. Visualization of the bisulfite sequencing results for *GBA3* and the PGC−1α promoters were completed using MethTools 3.0. **B.** Methylation sequencing region in the PGC−1α promoter. Important transcription factor binding sites are underlined. Methylated CpGs are highlighted in brown, CpAs in red, CpTs in green and CpCs in blue. **C**. Quantitation of cytosine methylation levels of the PGC−1α promoter. The percentage of cytosine methylation in the PGC−1α promoter is 1.52 in control and 2.43 in PD respectively, and they are significantly different (p = 0.0368). **D.** Methylation sequencing region in the *GBA3* promoter. Important transcription factor binding sites are underlined. Methylated CpGs are highlighted in brown. **E.** Quantitation of cytosine methylation levels of the *GBA3* promoter. The percentage of cytosine methylation in GBA3 promoter is 5.93 in control and 4.89 in PD respectively; they are not significantly different (p = 0.39). **F.** PGC-1α gene expression was determined by real time PCR. **G**. Image of representative western blot for PGC−1α protein expression in control and PD nigral samples. **H**. Quantitative densitometry of the western blotting analysis. The expression of PGC−1α was normalized for actin protein expression. **I.** Quantitation of the mitochondria DNA (mtDNA) to nuclear DNA (nuDNA) ratio using real-time PCR. Results were presented as mean±SEM (*p<0.05). Differences between groups were determined by unpaired Student’s t test.

To examine a potential mechanism for our previously reported down-regulation of PGC−1α in SN of sporadic PD, we analyzed the PGC−1α promoter for evidence of hypermethylation, a known epigenetic modification associated with decreased transcription. We performed DNA methylation analysis on SN tissue from sporadic PD and age-matched normal controls (**Table 1**). PD patients had been clinically diagnosed with progressive PD and manifested neuropathologic Lewy bodies and dopaminergic neuronal degeneration. The age-matched normal subjects were studied to control for age related changes in methylation (age: 78.9±7.03 for PD vs 77.7±8.98 for control) [27, 28]. Genomic DNA was isolated from SN and subjected to bisulfite sequencing. The sequenced region spanned -358 to -60 relative to the transcription start site (+1) of the PGC−1α promoter and included six major transcription factor binding sites (**Fig 1B**). We observed an increase in cytosine methylation in this region in PD patients compared to controls (**Fig 1C**). The sequencing results revealed that the majority of the methylated cytosines were not CpG dinucleotides, rather they were non-canonical cytosine methylated residues (**Fig 1A & 1B**). Quantitatively, there was an overall 1.6-fold increase in cytosine methylation of the PGC−1α promoter in PD patients compared with control subjects (p<0.05, **Fig 1C**). The most commonly methylated dinucleotide was CpT and the ratio of 5mCT to total C was increased from 0.34 (±0.18) in control to 0.74 (±0.27) in PD patients. To determine whether this hypermethylation in PD was a feature of the genomic locus harboring the PGC−1α gene, we examined an adjacent gene, *GBA3*, located more proximal to the telomere on chromosome 4. In PD patients the *GBA3* promoter region was not hypermethylated (**Fig 1A, 1D & 1E**), making evident that adjacent genes are differentially methylated in sporadic PD.

**Table 1:** Patient Demographics.

	Age (years)	Gender	PMI (h)
**Parkinson’s disease case no.**			
**1**	**79**	**F**	**34**
**2**	**72**	**M**	**3**
**3**	**75**	**F**	**19**
**4**	**82**	**F**	**18**
**5**	**76**	**M**	**19.3**
**6**	**73**	**M**	**22.2**
**7**	**93**	**F**	**33.3**
**8**	**72**	**M**	**30.7**
**9**	**79**	**M**	**38.6**
**10**	**88**	**M**	**26.5**
**Mean±SE**	**78.9±7.03**		**24.5±10.41**
**Control case no.**			
**1**	**73**	**F**	**13**
**2**	**68**	**M**	**18**
**3**	**75**	**F**	**24**
**4**	**81**	**M**	**17**
**5**	**71**	**M**	**26.2**
**6**	**77**	**M**	**14.4**
**7**	**95**	**F**	**9.7**
**8**	**68**	**F**	**19.1**
**9**	**79**	**F**	**17.8**
**10**	**90**	**M**	**7.2**
**Mean±SE**	**77.7±8.98**		**16.6±5.87**

Next we examined whether hypermethylation of the PGC−1α promoter was associated with down-regulation of PGC−1α expression at the transcript and protein levels. In PD patients, we observed a 53.7% decrease in PGC−1α gene expression compared to control subjects (p = 0.0108, **Fig 1F**), consistent with a prior meta-analysis of microarray data [1]. At the protein level, PGC−1α was decreased by 44.0% in PD relative to control (p = 0.046, **Fig 1G & 1H**). We also found that mitochondrial was significantly decreased in PD (**Fig 1I**).

We examine whether PGC−1α promoter hypermethylation in PD was accompanied by changes in makers of specific cell populations with the SN. We measured the gene expression of Tyrosine Hydroxylase (**TH**; dopaminergic neuronal marker), Neuronal Nuclei (**NeuN**; neuronal marker), Glial Fibrillary Acidic Protein (**GFAP**; astrocyte marker), and Ionized Calcium-binding Adapter Molecule 1 (**Iba1**; microglial marker) in PD and controls. Although TH gene expression was significantly decreased in PD SN (**S1A Fig**), indicative of dopaminergic neurodegeneration in PD patients, there were no difference in NeuN, GFAP and Iba1 gene expression between PD and control (**S1B–S1D Fig**).

We further sought to determine whether expression of the enzyme(s), DNMT3A, possibly responsible for the observed CpH (H = G/A/T/C) PGC−1α promoter methylation; in SN from PD compared to control subjects there was an insignificant but modest increase of DNMT3A (**S1E Fig**).

**Fig 2:**
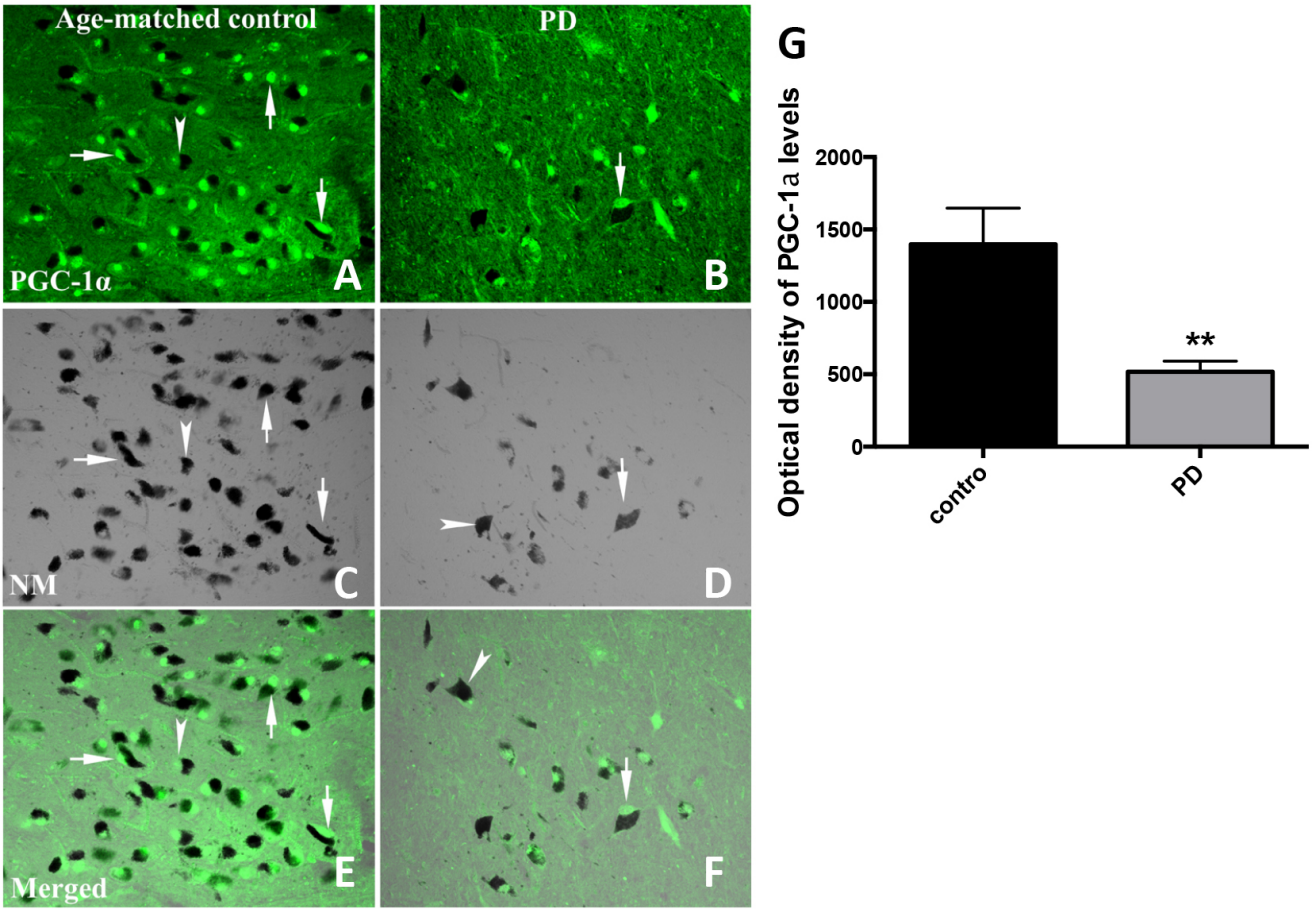
PGC−1α immunoreactivity in substantia nigra from PD and age-matched control subjects. PGC−1α immunofluorescent images were obtained with an Olympus confocal Fluoview microscope with a 20X magnification objective and a 488 nm excitation source for PGC−1α (**A** and **B**) and transparent optics for neuromelanin (NM; **C** and **D**). PGC−1α staining was shown with arrow(s) and NM staining with arrowheads. Optical density measurements of PGC−1α fluorescent density revealed that PGC−1α was significantly decreased compared to age-matched control subjects (**G**). Results presented as mean±SEM, **p<0.01. Differences between groups were determined by unpaired Student’s t test.

We next examined midbrain tissue from a separate group of PD and control subjects to determine whether differences in cellular PGC−1α protein were apparent. Using immunofluorescent labeling and optical density measurements we analyzed tissues from 12 subjects with sporadic PD (n = 6) or unaffected control subjects (n = 6). There were differences in age at the time of death (P<0.05) but not in post-mortem interval (P>0.05) between the two groups examined (**Table 2**). Details of these subjects were previously reported (Chu et al., 2006). PGC−1α immunoreactivity appeared to be concentrated in cell bodies (arrow), mainly localized in nuclei but some also displayed in perikarya (**Fig 2A and 2B**). In addition, the majority of PGC−1α staining was co-localized with neuromelanin (NM) (arrow head) (**Fig 2A–2D**), especially in age-matched control samples. NM provides an easily discernible endogenous marker for dopaminergic neurons, allowing for an assessment of co-localization with PGC−1α immunoreactive products in substantia nigra dopaminergic neurons. Optical density measurements within the substantia nigra revealed a significant decrease in the optical density of PGC−1α-immunoreactive signals in PD subjects compared to controls (**Fig 2G**). In conjunction with these findings, there was an evident decrease in the number of NM-containing nigral neurons in PD subjects relative to controls (**Fig 2C and 2D**)

**Table 2:** Patient Demographics.

	Age (years)	Gender	PMI (h)
**Parkinson’s disease case no.**			
**1**	**67**	**M**	**5.0**
**2**	**78**	**M**	**7.0**
**3**	**77**	**F**	**3.5**
**4**	**68**	**F**	**4.3**
**5**	**72**	**M**	**11.5**
**6**	**67**	**F**	**4.3**
**Mean±SE**	**71.50±2.04**		**5.93±1.21**
**Control case no.**			
**1**	**90**	**F**	**5.0**
**2**	**84**	**F**	**6.5**
**3**	**97**	**M**	**3.0**
**4**	**97**	**F**	**3.0**
**5**	**86**	**F**	**3.8**
**6**	**84**	**M**	**2.8**
**Mean±SE**	**89.67±2.48**		**4.01±0.59**

### Palmitate mediated hypermethylation of the PGC−1α promoter, reduced gene expression and reduced mitochondrial content in primary neurons

**Fig 3:**
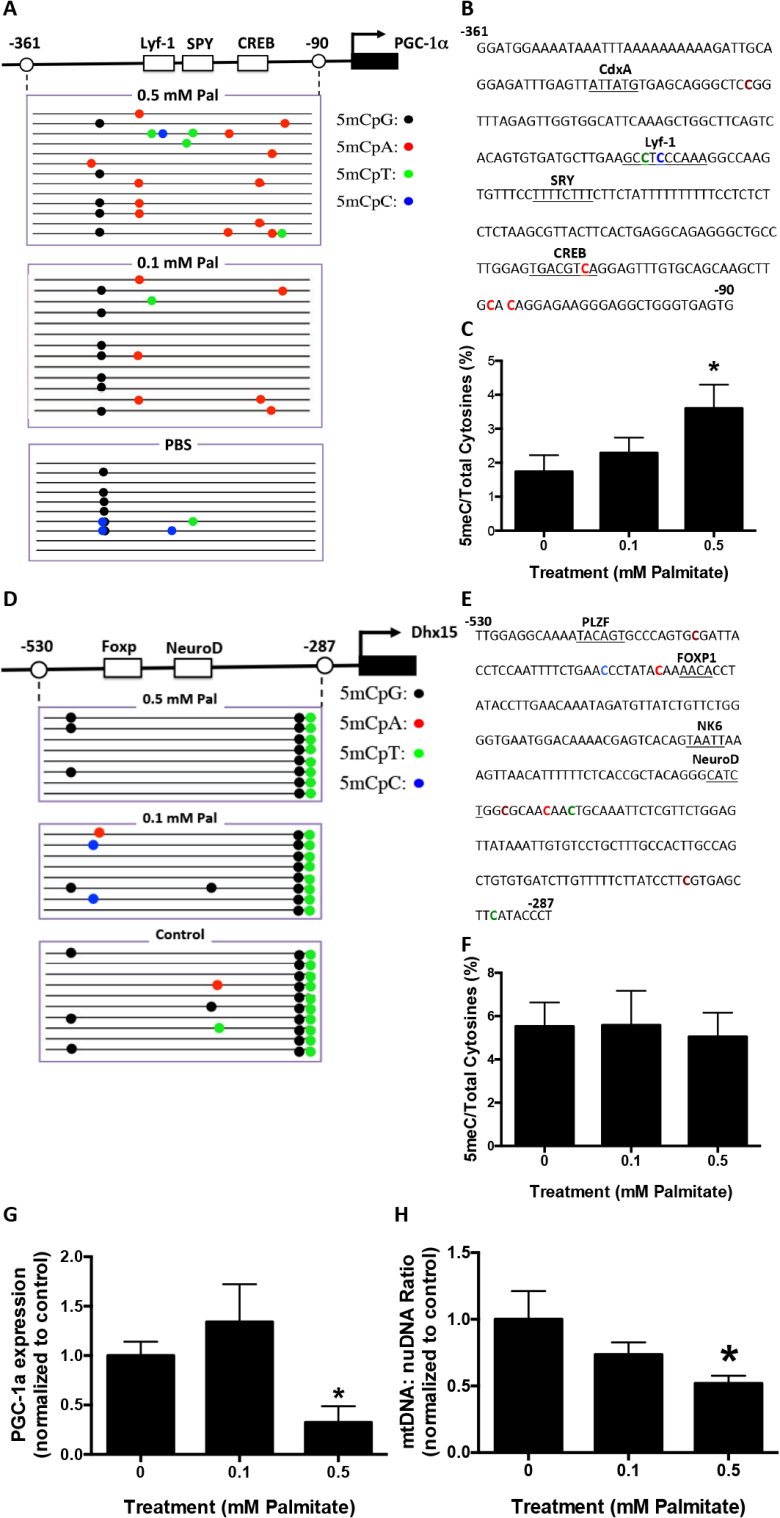
Palmitate induces PGC−1α promoter methylation in primary cortical neuronal cultures. Primary mouse cortical neurons were treated with 0.1 or 0.5 mM Palmitate (Pal) for 48 hours. Genomic DNA and mRNA were isolated for bisulfite sequencing and RT-PCR analysis, respectively. Visualization of the bisulfite sequencing results for the PGC−1α and *DHX15* promoters were completed using MethTools 3.0. **A**. Graphical depiction of the PGC−1α promoter region (-361 to -90) and location of methylated cytosines. **B.** Methylation sequencing region in the PGC−1α promoter. Important transcription factors are underlined. Methylated are CpG highlighted in brown, CpA in red, CpT in green and CpC in blue. **C.** Quantitation of cytosine methylation levels of the PGC−1α promoter. Increased methylation (106.9%) of PGC−1α promoter was observed in 0.5 mM palmitate-treated primary cortical neurons compared to PBS control (p = 0.02). **D.** Graphical depiction of the *DHX15* promoter region (-530 to -287) and location of methylated cytosines. **E**. Methylation sequencing region in the *DHX15* promoter. Important transcription factors are underlined. Methylated CpGs are highlighted in brown, CpAs in red, CpTs in green and CpCs in blue. **F.** Quantitation of cytosine methylation levels of *DHX15* promoter. **G**. Quantification of PGC−1α mRNA by qRT-PCR revealed a 72.0% decrease with 0.5 mM palmitate treatment compared with PBS controls (p = 0.0471). **H.** Quantitation of the mitochondria DNA (mtDNA) to nuclear DNA (nuDNA) ratio using real-time PCR. mtDNA:nuDNA ratio was decreased by 48.0% in 0.5 mM palmitate-treated cortical neuronal cultures compared to control (p = 0.0258). Results were presented as mean±SEM, *p<0.05. ANOVA with Student-Newman-Keuls post hoc analysis (C, F, G and H).

We examined whether the pro-inflammatory FFA, palmitic acid, would stimulate PGC−1α promoter hypermethylation, in primary murine neurons. Palmitate was added at 0.1 or 0.5 mM to cortical neuronal cultures for 48 hours. Genomic DNA was isolated and subjected to bisulfite sequencing which revealed that 0.5 mM palmitate mediated significant methylation of the promoter region (-361 to -90 relative to the cap site) of the PGC−1α gene (**Fig 3A & 3B**). The overall ratio of methylated to total cytosine residues was significantly increased with 0.5 mM palmitate (**Fig 3C**). The majority of methylated cytosines in 0.5 mM palmitate-treated cultures were non-canonical; CpA methylation was increased from 0 in control to 1.96 and CpT methylation was increased from 0.19 in control to 0.65 with palmitate treatment. One major locus for non-CpG methylation was located within the transcription factor cAMP response elements binding site (CREB), a *cis* regulatory element associated with PGC−1α transcription (**Fig 3B**). To determine whether palmitate mediated methylation was localized to the PGC−1α promoter, we analyzed the promoter region of *DHX15*, an adjacent gene on chromosome 5 in *Mus musculus*. No significant increase in *DHX15* cytosine methylation was measured in response to palmitate treatment (**Fig 3D to 3F**). To evaluate whether promoter hypermethylation affected gene expression, we measured PGC−1α transcript levels. A significant decrease of PGC−1α gene expression was observed in neuronal cultures treated with 0.5 mM palmitate (**Fig 3G**). In addition, the ratio of mtDNA to nuDNA was decreased in 0.5 mM palmitate-treated neuronal cultures (**Fig 3H**), indicative of reduced mitochondrial content.

### Palmitate mediated hypermethylation of the PGC−1α promoter, reduced gene expression and reduced mitochondrial content in primary glial cells

**Fig 4:**
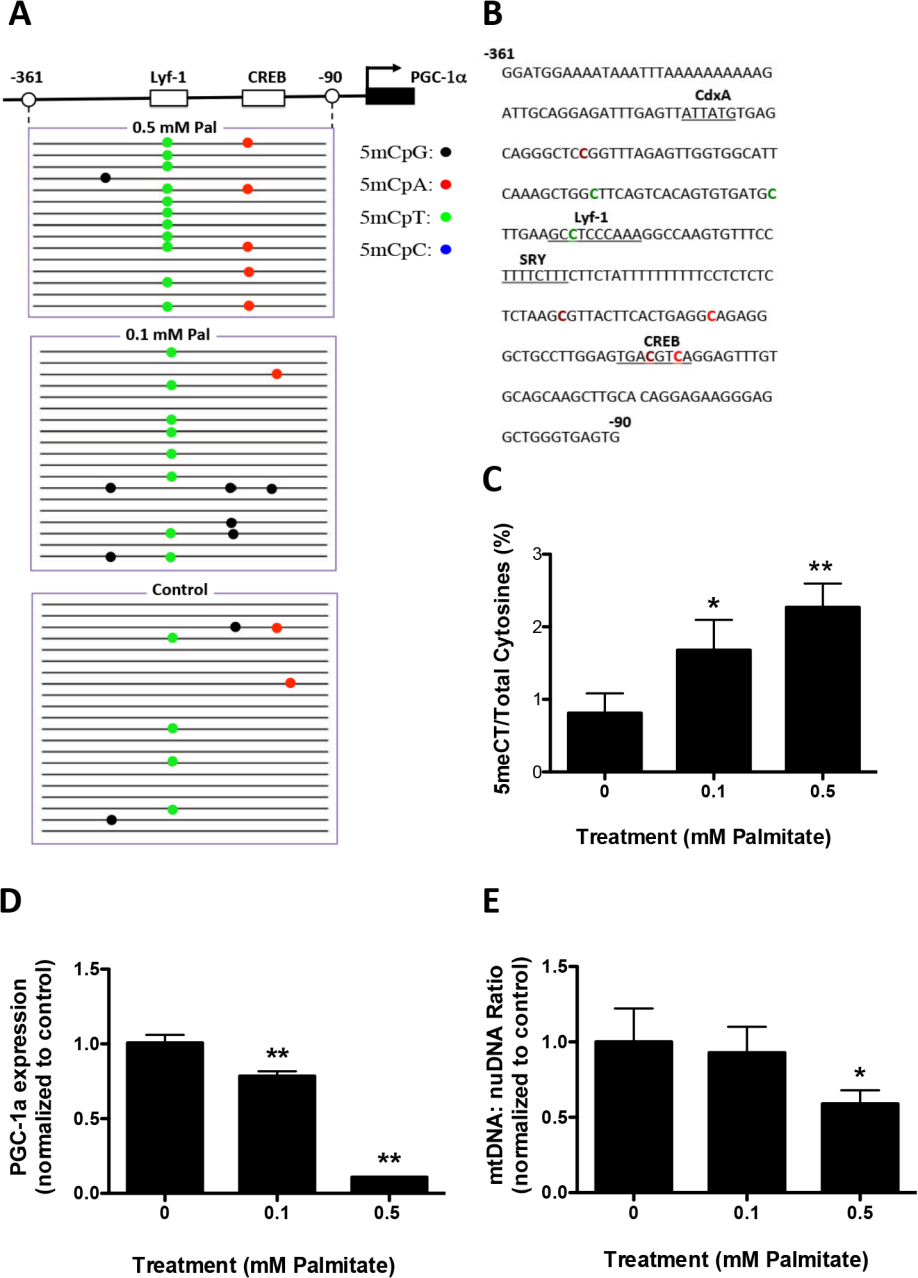
Palmitate induces PGC−1α promoter methylation in primary microglia. Primary microglia were treated with 0.1 or 0.5 mM Palmitate for 48 hours. Genomic DNA and mRNA were isolated for bisulfite sequencing and RT-PCR analysis. **A**. Graphical depiction of the PGC−1α promoter region (-361 to -90) and location of methylated cytosines. **B.** Methylation sequencing region in the PGC−1α promoter. Important transcription factors are underlined. Methylated CpGs are highlighted in brown, CpAs in red, CpTs in green and CpCs in blue. **C.** Quantitation of cytosine methylation levels of the PGC−1α promoter. Methylation of the PGC−1α promoter was increased by 107.4% with the treatment of 0.1 mM palmitate (p = 0.042) and 180.2% with the treatment of 0.5 mM palmitate (p = 0.0008) respectively compared to PBS control. **D.** Quantification of PGC−1α mRNA by qRT-PCR revealed a 21.6% decrease with the treatment of 0.1 mM palmitate (p = 0.0026) and 89.1% decrease with the treatment of 0.5 mM palmitate (p<0.0001) compared with PBS controls. **E.** Quantitation of mitochondria DNA (mtDNA) to nuclear DNA (nuDNA) ratio using real-time PCR. mtDNA:nuDNA ratio was decreased by 49.7% in 0.5 mM palmitate-treated microglial cultures compared to control (p = 0.046). Results presented as mean±SEM, *p<0.05, **<0.01, ANOVA with Student-Newman-Keuls post hoc analysis.

The down-regulation of PGC−1α gene expression in midbrain could occur in multiple cell types, including neurons and glial cells [2]. Microglia and astrocytes are two major groups of non-neuronal cells in CNS. We examined whether palmitate treatment would promote hypermethylation of the PGC−1α promoter in these cell types, leading to down-regulation of PGC−1α gene expression. Primary microglia or astrocytes were treated with 0.1 or 0.5 mM palmitate for 48 hours. Genomic DNA and total RNA were isolated, the former subjected to bisulfite sequencing and the latter to real-time PCR analysis. In microglia, both low (0.1 mM) and high (0.5 mM) concentrations of palmitate mediated significantly increased methylation of the PGC−1α promoter (**Fig 4A to 4C**). The most frequently methylated site was CpT within the transcriptional regulator Lymphocyte-specific DNA-binding protein (Lyf-1) binding site (**Fig 4B**). Similar to primary neuronal cultures, there was no significant increase in cytosine methylation of *DHX15* promoter in both microglia and astrocyte cultures (**S2 Fig**). Paralleling cytosine hypermethylation, both concentrations of palmitate reduced PGC−1α gene expression in microglia (**Fig 4D**). Only the high concentration of palmitate caused a significant decrease in mtDNA:nuDNA ratio (**Fig 4E**).

**Fig 5:**
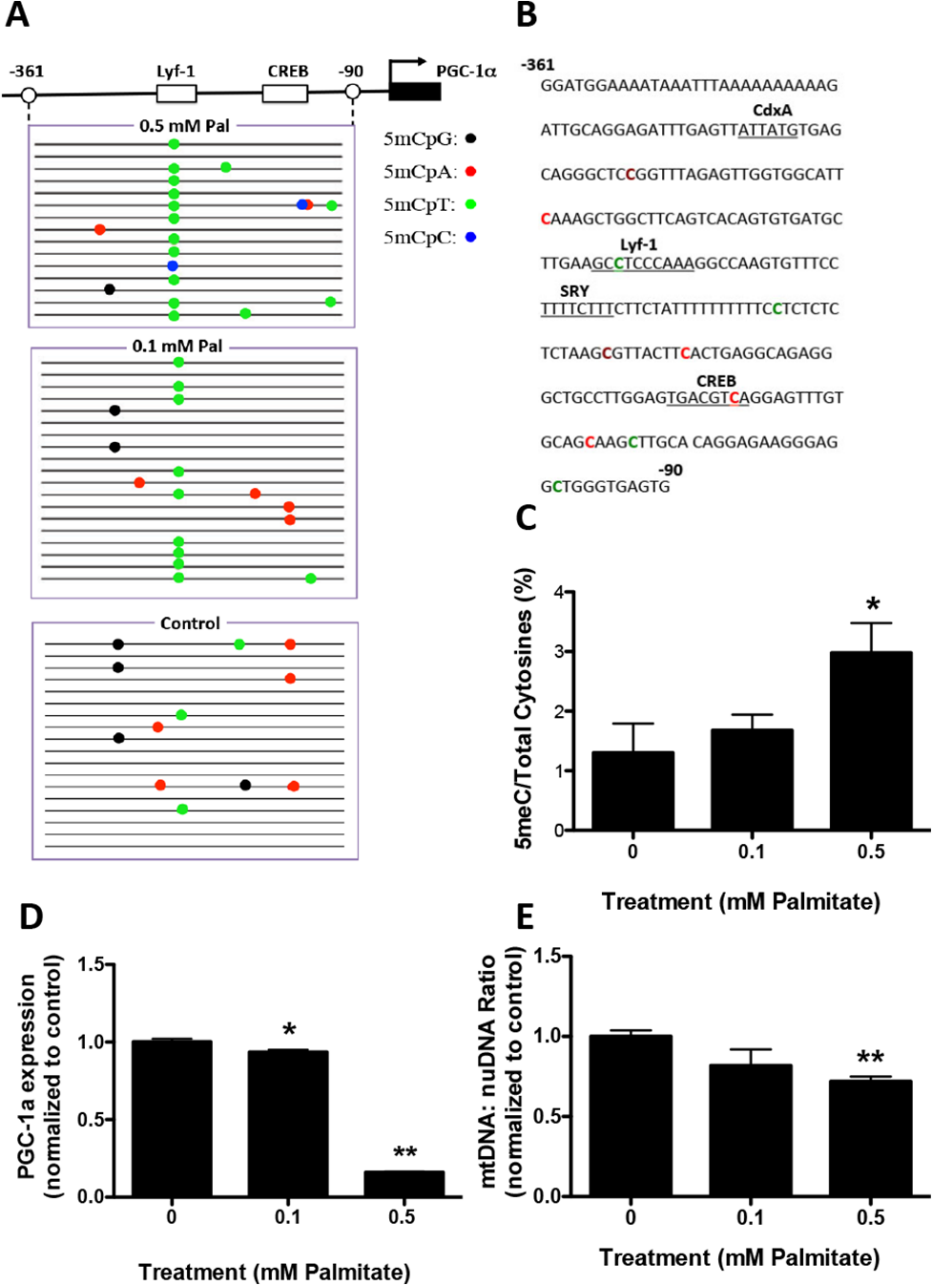
Palmitate induces PGC−1α promoter methylation in primary astrocytes. Primary astrocytes were treated with 0.1 or 0.5 mM Palmitate (Pal) for 48 hours. Genomic DNA and mRNA were isolated for bisulfite sequencing and RT-PCR analysis. **A**. Graphical depiction of the PGC−1α promoter region (-361 to -90) and location of methylated cytosines. **B.** Methylation sequencing region in the PGC−1α promoter. Important transcription factors are underlined. Methylated CpGs are highlighted in brown, CpAs in red, CpTs in green and CpCs in blue. **C.** Quantitation of cytosine methylation levels of the PGC−1α promoter. Methylation of the PGC−1α promoter was increased by 129.2% with the treatment of 0.5 mM palmitate (p = 0.012) compared to PBS control. **D.** Quantification of PGC−1α mRNA by qRT-PCR revealed a 6.4% decrease with the treatment of 0.1 mM palmitate (p = 0.0085) and 83.9% decrease with the treatment of 0.5 mM palmitate (p<0.0001) compared with PBS controls. **E.** Quantitation of the mitochondria DNA (mtDNA) to nuclear DNA (nuDNA) ratio using real-time PCR. mtDNA:nuDNA ratio was decreased by 28.1% in 0.5 mM palmitate-treated astrocyte cultures compared to control (p = 0.0006). Results presented as mean±SEM, *p<0.05, **<0.01, ANOVA with Student-Newman-Keuls post hoc analysis.

In primary astrocytes, the higher concentration of palmitate produced significantly increased methylation of the PGC−1α promoter (**Fig 5A to 5C**). Similar to primary microglia, the most frequently methylated site was CpT within the Lyf-1 binding site (**Fig 5B**). Low and high concentrations of palmitate both reduced PGC−1α gene expression (**Fig 5D)**. Only the high concentration of palmitate reduced mtDNA:nuDNA ratio (**Fig 5E**).

### Palmitate-mediated hypermethylation of the PGC−1α promoter in vivo

**Fig 6:**
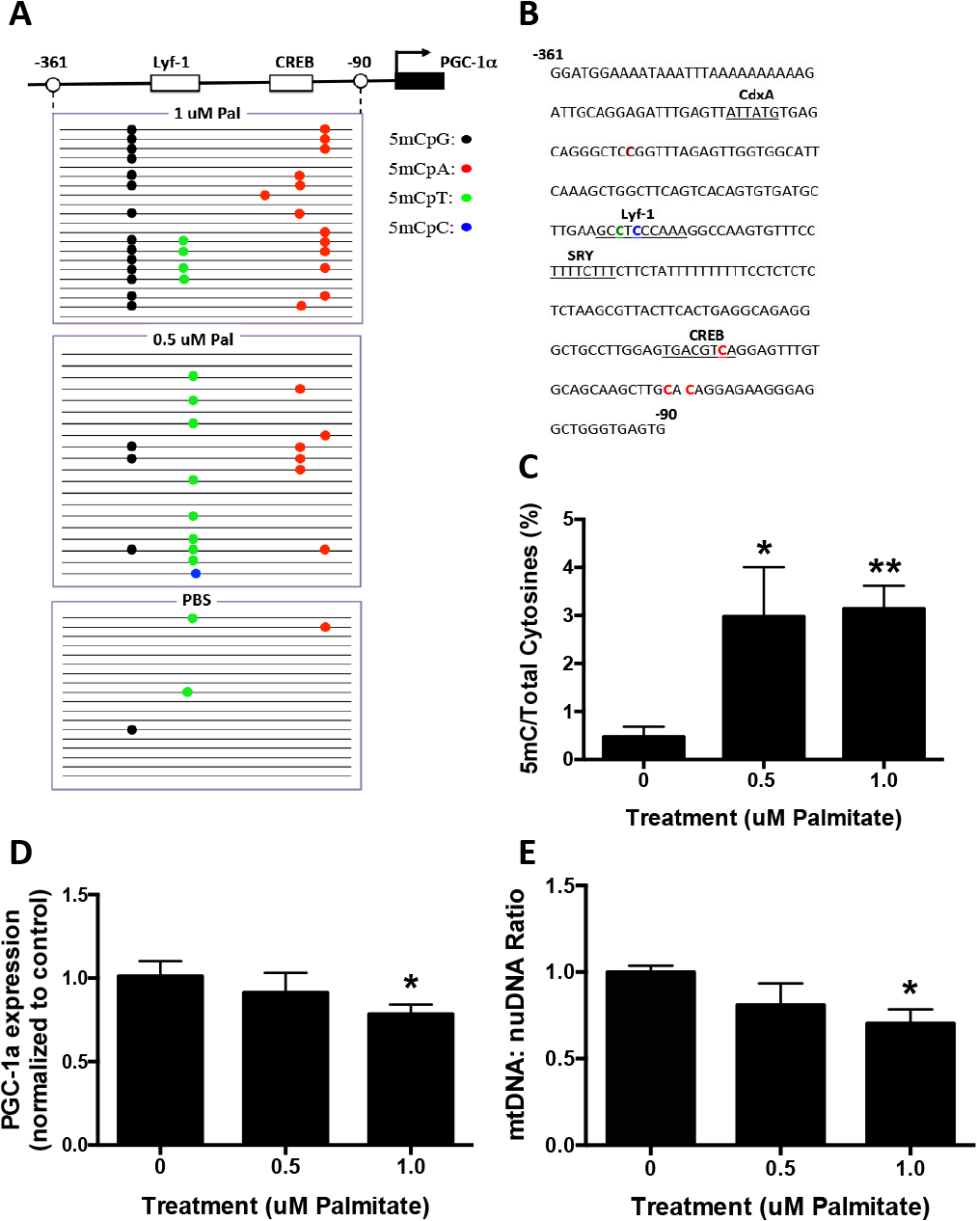
Palmitate induces PGC−1α promoter methylation in human mutant α−synuclein transgenic mice (DMSYN). Infusion pumps were implanted with a cannula aiming into the right lateral ventricle. 100uL of solution (PBS, 0.5 uM Pal or 1 uM Pal) were consistently delivered to the brain at a flow rate of 0.25uL/h for 14 days. **A**. Graphic representation of the bisulfite-sequenced portion (-361 to -90) of the PGC−1α promoter and visualization of bisulfite sequencing results as analyzed by MethTools 3.0. The transcription start site (arrow) and first exon (black box) are shown. **B**. Methylation sequencing region in the PGC−1α promoter. Important transcription factors are underlined. Methylated CpGs are highlighted in brown, CpAs in red, CpTs in green and CpCs in blue. **C.** Quantitation of cytosine methylation levels of the PGC−1α gene. Methylation of the PGC−1α promoter was increased by 630.1% with the treatment of 0.5 uM palmitate (p = 0.0294) and by 664.3% with the treatment of 1 uM palmitate (p<0.0001) compared to PBS control. **D**. Quantification of PGC−1α mRNA by qRT-PCR revealed a 21.7% decrease with the treatment of 1.0 uM palmitate (p = 0.038) compared with PBS controls. **E**. Quantitation of the mitochondria DNA (mtDNA) to nuclear DNA (nuDNA) ratio using real-time PCR. mtDNA:nuDNA ratio was decreased by 30.2% in 1.0 uM palmitate treatment compared to control (p = 0.02). Results were presented as mean±SEM, *p<0.05, **<0.01, ANOVA with Student-Newman-Keuls post hoc analysis.

To examine the *in vivo* effect of palmitate on PGC−1α promoter methylation we administered palmitate ICV to 6-month old mutant human α−synuclein transgenic mice (DMSYN). DMSYN transgenic mice express mutant α−synuclein (A30P and A53T) under the transcriptional control of the tyrosine hydroxylase promoter [23]. These transgenic mice manifest nigrostriatal axonal and terminal injury, age-related impairments in motor coordination and age-related reductions in dopamine and its metabolites. Recent evidence suggested that overexpression of α−synuclein, especially A53T, leads to enhanced lipid accumulation [29]. It is of great interest to evaluate the combined effects of external factors (diet, e.g., increased lipid uptake) and internal factors (inherited mutations) on disease initiation and progression, which, if successful, could lay the foundation for new animal models of PD. Accordingly, DMSYN transgenic mice received ICV palmitate and short-term effects were determined. DMSYN transgenic mice received 100 uL of 0.5, 1.0 uM palmitate, or PBS for 14 days, and then were euthanized. The treatment did not significantly affect body weight. Midbrain tissue was dissected and RNA and genomic DNA isolated from substantia nigra. Bisulfite sequencing revealed that palmitate increased PGC−1α promoter methylation at both low and high doses (**Fig 6A & 6C**). The two major methylated cytosine sites were CpT located within the Lyf-1 transcription factor binding site and CpA located within the CRE binding site (**Fig 6B**). High-dose palmitate caused significant decreases in PGC−1α gene expression (**Fig 6D**) and mitochondrial content (**Fig 6E**), while low dose palmitate only resulted in a slight decrease.

### Palmitate-mediated upregulation of the unfolded protein response and proinflammatory gene expression

**Fig 7:**
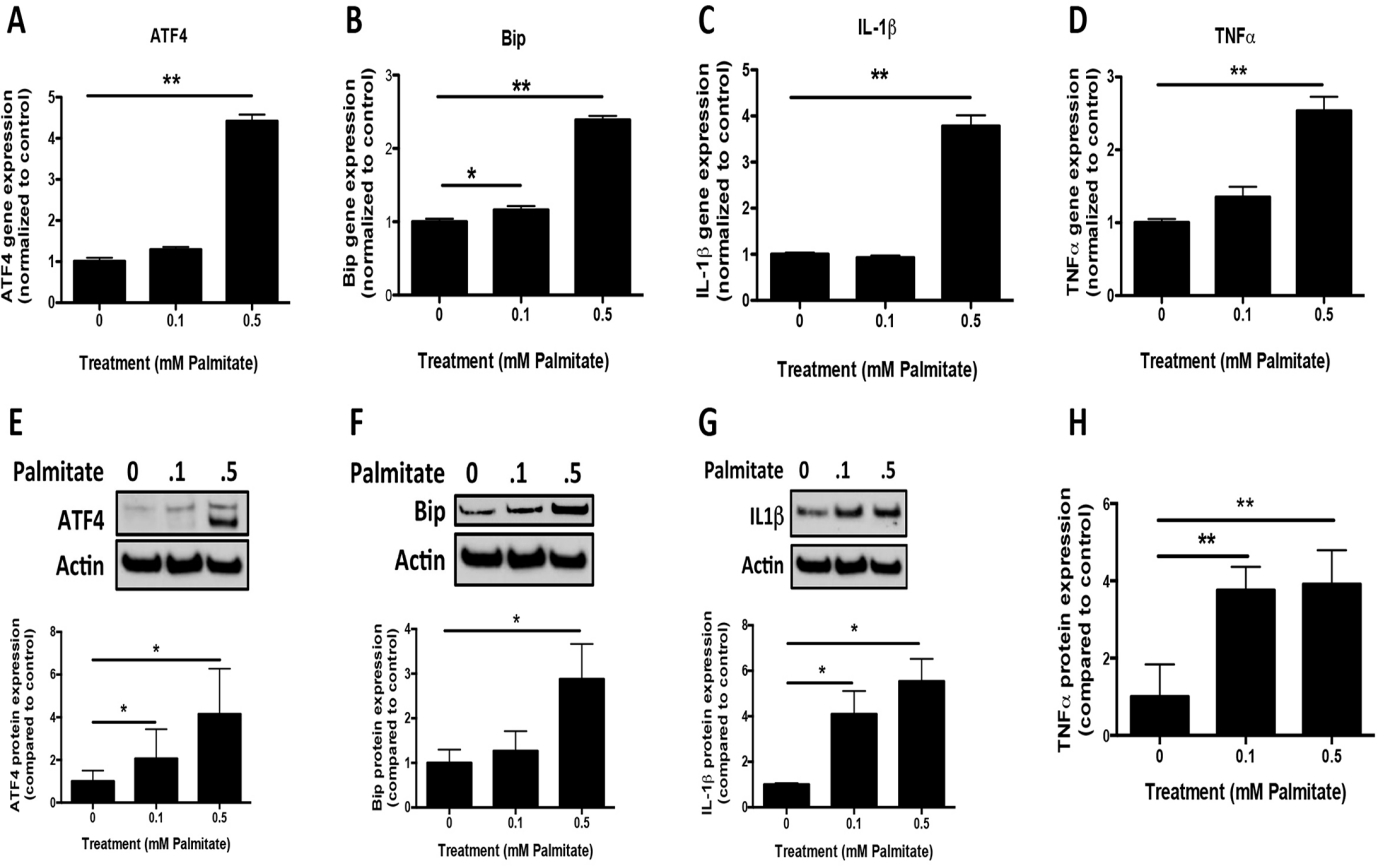
Unfolded protein response and proinflammatory gene expression profiling in primary astroyctes. Primary astrocytes were treated with 0.1 or 0.5 mM Palmitate (Pal) for 48 hours. mRNA and protein were isolated for RT-PCR, western blotting and ELISA analysis. UPR (ATF4 (**A**) and Bip (**B**)) and proinflammatory (IL1beta (**C**) and TNFα(**D**)) gene expression were measured by qRT-PCR in primary astrocytes. The protein level of ATF4 (**E**), Bip (**F**) and IL1beta (**G**) were determined by western blotting analysis. Quantification of western blot signals was normalized by actin expression and compared to control. **H**. ELISA analysis of TNFα secretion in the media of astrocyte culture with palmitate treatment. The quantification was compared to control (n = 4). Results were compared to control and presented as mean±SEM, *p<0.05, **<0.01, ANOVA with Student-Newman-Keuls post hoc analysis.

We next sought to identify potential upstream signaling events that may contribute to palmitate-mediated DNA methylation. Considering that primary astrocytes displayed robust responses to palmitate treatment *in vitro*, and GFAP gene expression was significantly increased in palmitate-treated DMSYN mice, it is likely that astrocytes play an important role in palmitate-mediated epigenetic alterations. Thus we focused on identifying upstream signaling events in astroyctes in this study. Previous studies showed that saturated FFA palmitate induced ER stress, inflammation, and insulin resistance in skeletal muscle cells [30]. To determine whether palmitate would also induce ER stress and/or an inflammatory response in astrocytes, we measured the expression of genes involved in the unfolded protein response (UPR), including ATF4 (activating transcription factor 4) and Bip (immunoglobulin heavy chain-binding protein) which are increased during ER stress, along with proinflammatory cytokines, including TNFα and IL1beta. We found that UPR genes ATF4 and Bip and proinflammatory genes IL1beta and TNFα were significantly increased in response to palmitate treatment (**Fig 7A to 7D**). We further confirmed the qRT-PCR gene expression results by western blotting and ELISA analysis. Using densitometric analyses we observed an increase of ATF4, Bip and IL1beta expression in a dose-dependent manner (**Fig 7E to 7G**), consistent with our gene expression data. TNFα secretion in the media of astrocyte cultures was robustly increased in cultures treated with both concentrations of palmitate (**Fig 7H**).

**Fig 8:**
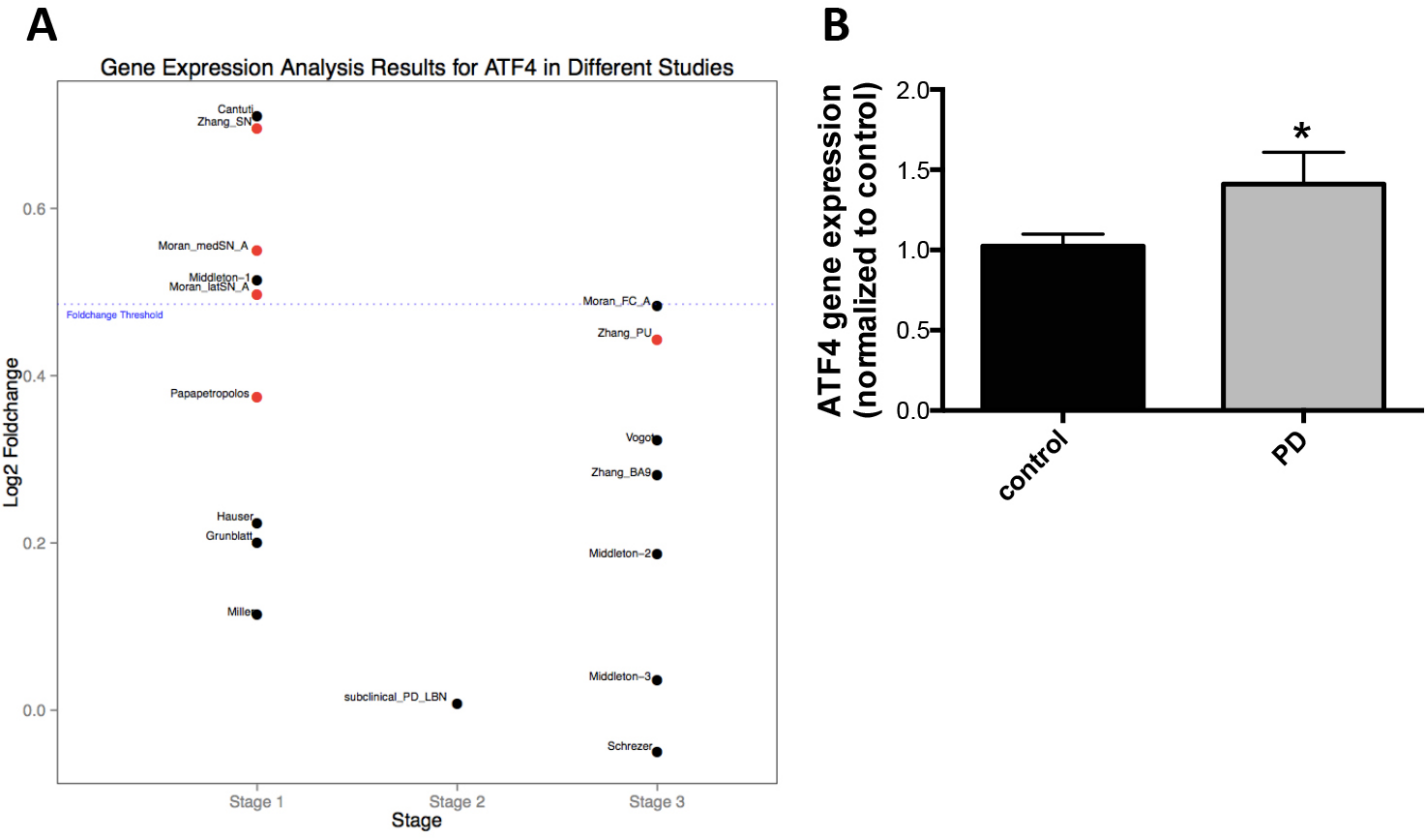
*ATF4* gene expression in microarray data sets with symptomatic Parkinson’s and subclinical disease and healthy controls and validation by qRT-PCR. **A.** 17 microarray datasets representing 221 PD patients and subclinical disease and 189 controls (GSE6613, GSE7621, GSE8397 (two data sets), GSE20141, GSE20146, GSE20153, GSE20159, GSE20163, GSE20164, GSE20168, GSE20291, GSE20292, GSE20314, GSE20333, and GSE24378; Gene Expression Omnibus) of human brain samples were used for differential gene expression analysis in this study. Stage 1 samples were from 185 microarrays (86 from controls and 99 from cases with symptomatic PD). Stage 2 samples were from 16 cases with subclinical, PD-related, α−synuclein positive, incidental lewy body disease (PD-LBN), and 17 age-, sex-, and postmortem interval matched controls without PD-related neuropathology on autopsy and also free of neurological disease. Stage 3 samples were from a total of 192 non-substantia nigra samples (106 from cases, 86 from controls without neurodegenerative disease). Data analysis was performed using R and Bioconductor packages. Each dataset was normalized separately using Robust Multi-array Average (RMA) implementation. Two-sample t-test with Storey method for false discovery rate (FDR) was carried out. The fold change threshold of PD/Normal, either up- or down-regulated, was at least 1.4-fold. The red dot indicated that the difference between two groups was statistically significant when p and q value were at least less than 0.05 and 0.25, respectively. B. ATF4 gene expression PD and control were subsequently measured by qRT-PCR (n = 10). Results were presented as mean±SEM. *p<0.05, differences between groups were determined by unpaired Student’s t test.

We extended these findings to PD patient substantia nigra tissue. We analyzed 17 microarray data sets from 221 patients with symptomatic sporadic PD and subclinical disease and 189 healthy controls [1]. We observed increased ATF4 gene expression in PD patients compared to control (fold change>1) in all data sets (**Fig 8A**). The fold changes for 5 data sets in stage 1 group (substantia nigra from symptomatic PD) was 1.4 (**Fig 8A**). We also analyzed ATF4 gene expression by qRT-PCR in patient samples that we used for our methylation analysis. Here we observed a significant increase in ATF4 gene expression in PD compared to control subjects (**Fig 8B**).

## Discussion

Our bisulfite analysis of human SN, primary neural cell cultures and mutant α−synuclein transgenic mice support an epigenetic mechanism for reducing transcription of the master mitochondrial regulator PGC−1α. We have demonstrated that non-canonical, i.e., non-CpG, methylation of the PGC−1α promoter occurred in all *in vivo* and cell culture systems. We further have shown that the FFA palmitate mediates the methylation of the PGC−1α promoter *in vitro* and *in vivo*. Our results provide a new framework to examine the role of epigenetic modification as a pathogenic contributor in sporadic human PD.

Human brain samples analysis indicated that PD was associated with increased methylation of the PGC−1α promoter and reduced expression of PGC−1α. PGC−1α positively regulates mitochondrial biogenesis and respiration, adaptive thermogenesis, glucogenesis and other metabolic processes [3]. Recent work highlighted a decrease in the expression of PGC−1α and downstream-regulated genes in the SN of PD, even in the earliest stages of PD [1, 2]. Our data also demonstrated a significant decrease of PGC−1α protein expression in two separate sets of PD samples compared to control. It should be noted that the ages of control samples in the fluorescent labeling study were more advanced than PD samples. As PGC−1α expression, along with mitochondrial functions, are reduced during telomere dysfunction, a condition associated with aging [31], we anticipate that more advanced age should be associated with further declines in PGC−1α gene expression. The emergence of PGC−1α as a key player in early PD raises the question as whether it is part of an etiologic mechanism.

How might we consider the significance of decreased PGC−1α expression in PD? Previous studies have shown that PGC−1α is required in neuronal cells for the induction of many ROS-detoxifying proteins, including glutathione peroxidase, catalase, UCP2 and SOD2. Diminished PGC−1α in mice exacerbates the neurodegenerative effects of MPTP [7]. In contrast, increased PGC−1α protects neurons from oxidative stress *in vitro*, a Syn-mediated cell death *in vitro*, and from MPTP-mediated neuronal degeneration *in vivo* [7, 32]. In addition, a long-term study using muscle-specific PGC−1α knockout mice demonstrates that loss of function causes age-dependent low-grade, chronic inflammation in white adipose and liver tissue [33]. Whether PGC−1α deficiency within the CNS, including both neuronal and glial cell compartments, will also be associated with age-dependent neuroinflammation remains uncertain but our preliminary studies suggest that PGC−1α knockout mice as well as knockout glial cultures are more susceptible to TLR4 agonist-induced neuroinflammation. Furthermore, enhanced astroglial PGC−1α levels markedly reduced the production and secretion of the proinflammatory mediators IL6 and chemokine ligand 2 [34]. Overall, PGC−1α down-regulation is associated with mitochondrial dysfunction, oxidative and inflammatory stress that may contribute to PD initiation and progression.

Our studies have shown that the PGC−1α promoter is hypermethylated and PGC−1α gene expression is down-regulated in the nigral samples from PD patients as well as α−synuclein transgenic mice treated with palmitate. Given that multiple cell types are present in the brain it is important to understand which cell types participate in dysregulation of the PGC−1α gene in PD. Our dissociated cell culture experiments demonstrate that palmitate exerts epigenetic effects on both neurons and glial cells (astrocytes and microglia) leading to distinct DNA methylation patterns. In primary neuronal cultures, DNA methylation mostly occurs at CpA sites, while in glial cells CpT methylation are more frequent. Interestingly, DNA methylation in the nigral tissues of PD patients and palmitate treated α−synuclein transgenic mice display a mixed pattern of dinucleotide methylation, where CpA and CpT occur to a similar extent, suggesting that both neurons and glial cells contribute in the substantia nigra. Additionally, in the α−synuclein transgenic mice treated with palmitate, there were no significant changes in TH and Iba1 gene expression (not shown) while GFAP was increased in the animals with high dose of palmitate treatment. These data suggest that the populations of TH positive neurons and Iba1 positive microglia do not appear to change significantly after 14-day palmitate treatment while increased numbers of GFAP positive astrocytes reflect reactivation in response to palmitate. The precise changes in the nigral cell populations *in vivo* remain to be determined by immunohistochemistry. Nevertheless, extant data allow us to posit that both neurons and glial cells contribute altered methylation of the PGC−1α promoter in patient and mouse brain; however glial cells, especially astrocytes, may be a significant contributor. Decades of pathological and physiological studies have focused on neuronal abnormalities in the neurodegenerative diseases, including PD, but increasingly, glial activation or alterations are recognized as an important feature of these neurodegenerative diseases. It is evident that activation of glial cells occurs prior to neuronal cell death and it may play an active role in the pathogenesis of the diseases [35]. Alterations or loss of normal function of glial cells may lead to a milieu harmful for dopaminergic neurons. Furthermore, given typically middle age onset of PD and the gradual loss of dopaminergic neurons in PD, a small epigenetic change to the PGC−1α gene in neurons accompanied with the reactive glial cell changes may antedate clinical diagnosis.

DNA methylation in the nervous system has been traditionally considered restricted to CpG dinucleotides. However, recent global profiling studies have revealed CpH (H = A/C/T) methylation in the adult mouse cortex [36, 37], adult mouse dentate neurons [38], and human brain [39]. In our study, we observed CpH methylation of the PGC−1α promoter in PD substantia nigra as well as palmitate-treated primary CNS cells and *in vivo* mouse substantia nigra of a PD transgenic model. These observations raise several important questions. First, does CpH methylation affect transcriptional regulation in mammalian cells? It is widely accepted that DNA methylation in promoter regions is linked to gene repression [40]. DNA methylation at promoter regions can attenuate transcription factor recruitment, resulting in transcriptional repression [41]. We found that the most frequently methylated cytosines were located in the regions of transcription factor binding sites CREB and Lyf-1. Whether DNA CpH methylation within or adjacent to these *cis* elements affect binding of cognate transcription factors remains to be investigated. However, our demonstration of CpH methylation of the PGC−1α promoter and down-regulation of PGC−1α expression strongly suggests methylation dependent gene silencing.

A second question is what enzyme(s) is responsible for the observed CpH PGC−1α promoter methylation. DNA methylation is catalyzed by a group of DNA methyltransferases (DNMTs). The eukaryotic DNMT family includes DNMT1, DNMT3A, DNMT3B and DNMT3L. DNMT1 is considered as a maintenance methyl transferase and predominantly methylates CpG dinuclotides [42]. DNMT3A and DNMT3B comprise the two major *de novo* methyl transferases. DNMT1 and DNMT3A (but not DNMT3B) are expressed at high levels in post-mitotic neurons [43]. In addition, DNMT3A protein is strongly expressed in rodent oligodendrocytes, a subset of astrocytes [44] and microglia [45]. A recent study has shown that DNMT3A knockdown led to a significant reduction in CpH, but not CpG, methylation, whereas DNMT1 knockdown showed little effect [38]. Collectively, these findings point to DNMT3A as involved in CpH methylation in the CNS. In depth studies will be required to dissect the potential role of DNMT3A or other DNMT in PD models.

In this study, we have demonstrated that treatment with palmitate causes hypermethylation of the PGC−1α promoter in cultured primary CNS cells and *in vivo*. Increasing evidence implicates saturated FFAs as pathogenic contributors in PD [12–18]. However, the proximate mechanism underlying palmitate-mediated alterations of the epigenome is not understood. Our finding that palmitate-mediated hypermethylation of PGC−1α promoter leads to down-regulation of PGC−1α gene expression provides a plausible mechanism that links a saturated FFA to PD pathogenesis. The pathobiology we postulate in PD follows: FFAs promote PGC−1α promoter methylation, a decline in PGC−1α levels and consequent down-regulation of an array of genes involved in energy metabolism, mitochondrial biogenesis [46–48], respiratory function [49], anti-inflammation [7, 50] and anti-oxidant defense [7, 51]. Diminished PGC−1α function is strongly predicted to cause dysfunction and death of neurons in an inflammatory milieu associated with excessive oxidative stress. The cumulative toxicity owing to reduced PGC−1α levels may be central to sporadic PD pathogenesis.

We provide the first evidence that a FFA induces hypermethylation and down-regulation of a key multifunctional gene in the CNS of sporadic PD patients, PD transgenic mice and in the three major resident cells of brain. However, other contributing mechanisms may also underlie PGC−1α down-regulation. Deficiency of Parkin, an E3 ligase, leads to down-regulation of PGC−1α through transcriptional repression mediated by PARIS [52]. Palmitate reduces phosphorylation of AMP-activated protein kinase (AMPK) [30], which can affect PGC−1α expression and activity in muscle [53] and adipose tissues [54]. Future work is required to delineate mechanism(s) and the magnitude of their respective contribution(s) to PGC−1α promoter methylation and/or decreased PGC−1alphha gene expression.

We also generated a new PD mouse model, one combining both epigenetic and genetic components. Sporadic PD is an etiologically heterogeneous disease with genetic influences. Epigenetic modifications could provide a potential linkage between environmental effects and genetic susceptibilities and thus increase risk for certain PD-associated genetic polymorphisms. Nutrition, chemical exposures, and extrinsic physical factors all could affect disease risk through epigenetic mechanisms. Herein we show that palmitate induces epigenetic change in α−synuclein transgenic mice. One common neuropathological feature of PD is the accumulation of α−synuclein in a subset of vulnerable neurons [55], which provides a rationale for the use of this transgenic model. α−synuclein transgenic mice express a mutant form of human α−synuclein (A30T & A53T) and display a progressive neurodegenerative process characterized by loss of SN TH+ neuron by ~20 months of age [23]. In combination with the transgene, we have added an epigenetic component to drives neurodegeneration. Following 14-days of palmitate infusion, we observed hypermethylation in the PGC−1α promoter and decreased PGC−1α gene expression and mitochondrial content. We also observed an increase in GFAP gene expression. However, we did not detect a significant decrease in TH gene expression in response to palmitate at this time point (not shown). Collectively our *in vivo* data suggest that short-term infusion of palmitate mediates epigenetic effects along with neuroinflammation but without neurodegeneration; this portends a model whereby one can separate and study independently the effects of gene and environmental interactions. Given that epigenetic modification of the PGC−1α promoter was accompanied by decreased PGC−1α gene expression and mitochondrial content, we postulate palmitate enhances susceptibility of the vulnerable dopaminergic neurons. There is a need for new animal models of PD that more faithfully recapitulate PD and which afford the potential to study clinically relevant environmental modulators. Whether our combined α−synuclein/palmitate mouse model will address this needs merits further investigation.

**Fig 9:**
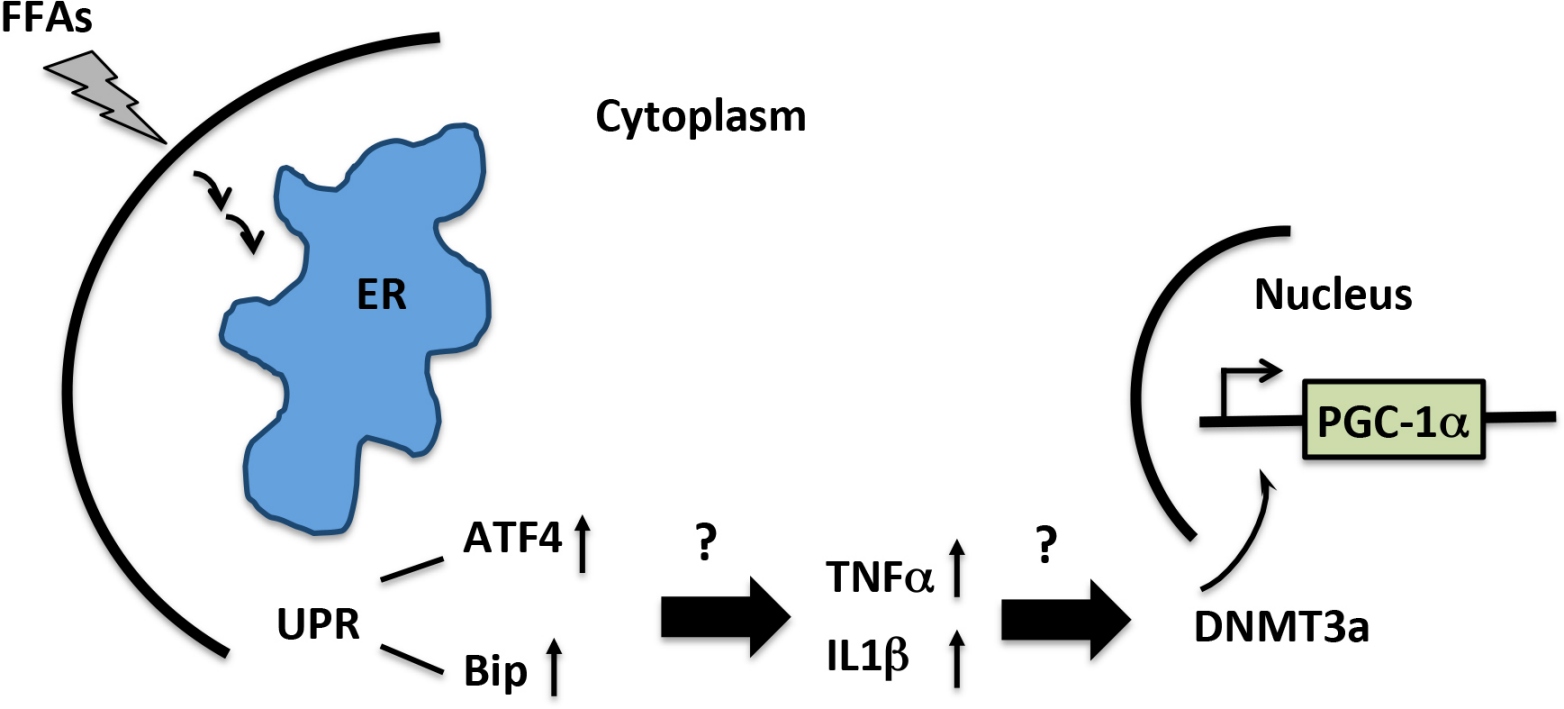
Diagrammatic illustration of a possible mechanism underlying FFA-mediated epigenetic modification of the PGC−1α promoter in astrocytes. We hypothesize that palmitate induces epigenetic modification of the PGC−1α promoter by activating the UPR (ATF4 and Bip) measured by increased gene expression and/or subsequent inflammatory gene expression in CNS. The UPR or inflammatory responses mediate recruitment of DNA methytransferases such as DNMT3A to nucleus to catalyze methylation of the PGC−1α promoter.

We present evidence that a FFA induces upregulation of the UPR and pro-inflammatory gene expression in primary astrocytes. These findings align with previous studies showing that palmitate induces ER stress, inflammation, and insulin resistance in skeletal muscle cells [30]. Moreover, we observed an increase of the UPR gene ATF4 in SN in sporadic PD patients compared to control subjects. The mechanisms underlying palmitate induced ER stress in CNS cell types are unresolved. One study has shown that palmitate stimulated ER stress via protein palmitoylation in SH-SY5Y cells [56]. Since ER is sensitive to aberrant proteins, uncontrolled protein palmitoylation could exacerbate ER stress [57]. Previous studies have shown that UPR induces hypermethylation of cystic fibrosis transmembrane conductance regulator (CFTR) promoter and leads to gene repression [58]. ER stress and the UPR pathway modulate inflammation through the upregulation of pro-inflammatory genes such as TNFα [59, 60]. The pro-inflammatory cytokine TNFα can induce methylation of the PGC−1α promoter in muscle cells [61]. Collectively, these results suggest a novel mechanism through which a FFA mediates epigenetic silencing of the PGC−1α gene perhaps by activating the UPR and pro-inflammatory gene expression in astrocytes (**Fig 9**). It is of great interest to determine the upstream signaling pathways in neurons as well. Given the distinct methylation pattern in neurons, palmitate may exert a distinct signaling pathway in neurons in contrast to glial cells.

In summary, our study provides a new framework to link FFA, epigenetic methylation of the PGC−1α promoter, diminished PGC−1α gene expression, reduced mitochondrial content and consequent bioenergetics compromise. It is vital to establish whether dietary or other environmental factors can be accommodated in this framework. Finally, it is interest to determine whether PGC−1α promoter methylation can be reversed by therapeutics, either through targeting ER stress or inflammation signaling pathways, and whether restoration of PGC−1α expression will be neuroprotective.

## Supporting Information

S1 Fig Gene expression profiling in PD patients and age-matched controls. CNS cell markers TH (A), NeuN (B), GFAP (C) and Iba1 (D) gene expression was determined by real time PCR. **E**. DNMT3A gene expression was measured by real time PCR. Results were presented as mean±SEM (*p<0.05). Differences between groups were determined by unpaired Student’s t test.
(TIF)

S2 Fig Palmitate does not induce *DHX15* promoter methylation in primary microglia and astrocytes. Primary microglia and astrocytes were treated with 0.1 and 0.5 mM Palmitate for 48 hours. Genomic DNA was isolated for bisulfite sequencing. Visualization of the bisulfite sequencing results for *DHX15* promoter was completed using MethTools 3.0. **A**. Graphical depiction of the *DHX15* promoter region (-530 to -287) and location of methylated ctyosines in primary microglia. **B.** Methylation sequencing region in the *DHX15* promoter in primary microglia. Important transcription factors are underlined. Methylated CpGs are highlighted in brown, CpAs in red, CpTs in green and CpCs in blue. **C.** Quantitation of cytosine methylation levels of *DHX15* promoter in primary microglia. **D**. Graphical depiction of the DHX15 promoter region (-530 to -287) and location of methylated ctyosines in primary astrocytes. **E.** Methylation region sequenced in the *DHX15* promoter. Important transcription factors are underlined. Methylated CpGs are highlighted in brown, CpAs in red, CpTs in green and CpCs in blue. **F.** Quantitation of cytosine methylation levels of *DHX15* promoter in primary astrocytes. Results were presented as mean±SEM, ANOVA with Student-Newman-Keuls post hoc analysis (C and F).
(TIF)

## References

[1] Zheng B, Liao Z, Locascio JJ, Lesniak KA, Roderick SS, Watt ML, et al PGC-1α, a potential therapeutic target for early intervention in Parkinson's disease. Sci Transl Med. 2010;2(52):52ra73 Epub 2010/10/12. 10.1126/scitranslmed.3001059 20926834 PMC3129986

[2] Eschbach J, von Einem B, Muller K, Bayer H, Scheffold A, Morrison BE, et al Mutual exacerbation of peroxisome proliferator-activated receptor gamma coactivator 1α deregulation and α-synuclein oligomerization. Annals of neurology. 2015;77(1):15–32. 10.1002/ana.24294 25363075 PMC4293280

[3] Handschin C, Spiegelman BM. Peroxisome proliferator-activated receptor gamma coactivator 1 coactivators, energy homeostasis, and metabolism. Endocrine reviews. 2006;27(7):728–35. 10.1210/er.2006-0037 . 17018837

[4] Lin J, Handschin C, Spiegelman BM. Metabolic control through the PGC-1 family of transcription coactivators. Cell metabolism. 2005;1(6):361–70. 10.1016/j.cmet.2005.05.004 . 16054085

[5] Soyal S, Krempler F, Oberkofler H, Patsch W. PGC-1α: a potent transcriptional cofactor involved in the pathogenesis of type 2 diabetes. Diabetologia. 2006;49(7):1477–88. 10.1007/s00125-006-0268-6 . 16752166

[6] Cui L, Jeong H, Borovecki F, Parkhurst CN, Tanese N, Krainc D. Transcriptional repression of PGC-1α by mutant huntingtin leads to mitochondrial dysfunction and neurodegeneration. Cell. 2006;127(1):59–69. Epub 2006/10/05. 10.1016/j.cell.2006.09.015 . 17018277

[7] St-Pierre J, Drori S, Uldry M, Silvaggi JM, Rhee J, Jager S, et al Suppression of reactive oxygen species and neurodegeneration by the PGC-1 transcriptional coactivators. Cell. 2006;127(2):397–408. Epub 2006/10/24. 10.1016/j.cell.2006.09.024 . 17055439

[8] Weydt P, Pineda VV, Torrence AE, Libby RT, Satterfield TF, Lazarowski ER, et al Thermoregulatory and metabolic defects in Huntington's disease transgenic mice implicate PGC-1α in Huntington's disease neurodegeneration. Cell metabolism. 2006;4(5):349–62. Epub 2006/10/24. 10.1016/j.cmet.2006.10.004 . 17055784

[9] Parker WD Jr., Boyson SJ, Parks JK. Abnormalities of the electron transport chain in idiopathic Parkinson's disease. Annals of neurology. 1989;26(6):719–23. Epub 1989/12/01. 10.1002/ana.410260606 . 2557792

[10] Janetzky B, Hauck S, Youdim MB, Riederer P, Jellinger K, Pantucek F, et al Unaltered aconitase activity, but decreased complex I activity in substantia nigra pars compacta of patients with Parkinson's disease. Neurosci Lett. 1994;169(1–2):126–8. Epub 1994/03/14. . 8047266 10.1016/0304-3940(94)90372-7

[11] Burdge GC, Lillycrop KA. Fatty acids and epigenetics. Curr Opin Clin Nutr Metab Care. 2014;17(2):156–61. Epub 2013/12/11. 10.1097/MCO.0000000000000023 . 24322369

[12] Logroscino G, Marder K, Cote L, Tang MX, Shea S, Mayeux R. Dietary lipids and antioxidants in Parkinson's disease: a population-based, case-control study. Annals of neurology. 1996;39(1):89–94. Epub 1996/01/01. 10.1002/ana.410390113 . 8572672

[13] Chen H, Zhang SM, Hernan MA, Willett WC, Ascherio A. Dietary intakes of fat and risk of Parkinson's disease. Am J Epidemiol. 2003;157(11):1007–14. Epub 2003/06/05. . 12777364 10.1093/aje/kwg073

[14] Morris JK, Bomhoff GL, Stanford JA, Geiger PC. Neurodegeneration in an animal model of Parkinson's disease is exacerbated by a high-fat diet. Am J Physiol Regul Integr Comp Physiol. 2010;299(4):R1082–90. Epub 2010/08/13. 10.1152/ajpregu.00449.2010 20702796 PMC2957375

[15] Lu M, Hu G. Targeting metabolic inflammation in Parkinson's disease: implications for prospective therapeutic strategies. Clin Exp Pharmacol Physiol. 2012;39(6):577–85. Epub 2011/12/01. 10.1111/j.1440-1681.2011.05650.x . 22126374

[16] Santiago JA, Potashkin JA. Shared dysregulated pathways lead to Parkinson's disease and diabetes. Trends Mol Med. 2013;19(3):176–86. Epub 2013/02/05. 10.1016/j.molmed.2013.01.002 . 23375873

[17] Hu G, Jousilahti P, Bidel S, Antikainen R, Tuomilehto J. Type 2 diabetes and the risk of Parkinson's disease. Diabetes Care. 2007;30(4):842–7. Epub 2007/01/26. 10.2337/dc06-2011 . 17251276

[18] Qiu C, Hu G, Kivipelto M, Laatikainen T, Antikainen R, Fratiglioni L, et al Association of blood pressure and hypertension with the risk of Parkinson disease: the National FINRISK Study. Hypertension. 2011;57(6):1094–100. Epub 2011/05/04. 10.1161/HYPERTENSIONAHA.111.171249 . 21536985

[19] Jafari S, Etminan M, Aminzadeh F, Samii A. Head injury and risk of Parkinson disease: a systematic review and meta-analysis. Mov Disord. 2013;28(9):1222–9. Epub 2013/04/24. 10.1002/mds.25458 . 23609436

[20] Homayoun P, Rodriguez de Turco EB, Parkins NE, Lane DC, Soblosky J, Carey ME, et al Delayed phospholipid degradation in rat brain after traumatic brain injury. J Neurochem. 1997;69(1):199–205. Epub 1997/07/01. . 9202311 10.1046/j.1471-4159.1997.69010199.x

[21] Nicolai E, Cuocolo A, Acampa W, Varrone A, Pace L, Salvatore M. Exercise-test Tc-99m tetrofosmin SPECT in patients with chronic ischemic left ventricular dysfunction: direct comparison with Ti-201 reinjection. J Nucl Cardiol. 1999;6(3):270–7. Epub 1999/06/29. . 10385182 10.1016/s1071-3581(99)90038-7

[22] Karmi A, Iozzo P, Viljanen A, Hirvonen J, Fielding BA, Virtanen K, et al Increased brain fatty acid uptake in metabolic syndrome. Diabetes. 2010;59(9):2171–7. Epub 2010/06/23. 10.2337/db09-0138 20566663 PMC2927939

[23] Richfield EK, Thiruchelvam MJ, Cory-Slechta DA, Wuertzer C, Gainetdinov RR, Caron MG, et al Behavioral and neurochemical effects of wild-type and mutated human α-synuclein in transgenic mice. Exp Neurol. 2002/05/16 ed2002. p. 35–48. 12009758 10.1006/exnr.2002.7882

[24] Grunau C, Schattevoy R, Mache N, Rosenthal A. MethTools—a toolbox to visualize and analyze DNA methylation data. Nucleic Acids Res. 2000;28(5):1053–8. Epub 2000/02/10. 10666443 10.1093/nar/28.5.1053 PMC102603

[25] Gentleman RC, Carey VJ, Bates DM, Bolstad B, Dettling M, Dudoit S, et al Bioconductor: open software development for computational biology and bioinformatics. Genome Biol. 2004;5(10):R80 10.1186/gb-2004-5-10-r80 15461798 PMC545600

[26] Irizarry RA, Hobbs B, Collin F, Beazer-Barclay YD, Antonellis KJ, Scherf U, et al Exploration, normalization, and summaries of high density oligonucleotide array probe level data. Biostatistics. 2003;4(2):249–64. 10.1093/biostatistics/4.2.249 . 12925520

[27] Siegmund KD, Connor CM, Campan M, Long TI, Weisenberger DJ, Biniszkiewicz D, et al DNA methylation in the human cerebral cortex is dynamically regulated throughout the life span and involves differentiated neurons. PLoS One. 2007;2(9):e895 Epub 2007/09/20. 10.1371/journal.pone.0000895 17878930 PMC1964879

[28] Bjornsson HT, Sigurdsson MI, Fallin MD, Irizarry RA, Aspelund T, Cui H, et al Intra-individual change over time in DNA methylation with familial clustering. JAMA. 2008;299(24):2877–83. Epub 2008/06/26. 10.1001/jama.299.24.2877 18577732 PMC2581898

[29] Outeiro TF, Lindquist S. Yeast cells provide insight into α-synuclein biology and pathobiology. Science. 2003;302(5651):1772–5. Epub 2003/12/06. 10.1126/science.1090439 14657500 PMC1780172

[30] Salvado L, Coll T, Gomez-Foix AM, Salmeron E, Barroso E, Palomer X, et al Oleate prevents saturated-fatty-acid-induced ER stress, inflammation and insulin resistance in skeletal muscle cells through an AMPK-dependent mechanism. Diabetologia. 2013;56(6):1372–82. 10.1007/s00125-013-2867-3 . 23460021

[31] Sahin E, Colla S, Liesa M, Moslehi J, Muller FL, Guo M, et al Telomere dysfunction induces metabolic and mitochondrial compromise. Nature. 2011;470(7334):359–65. 10.1038/nature09787 21307849 PMC3741661

[32] Mudo G, Makela J, Di Liberto V, Tselykh TV, Olivieri M, Piepponen P, et al Transgenic expression and activation of PGC-1α protect dopaminergic neurons in the MPTP mouse model of Parkinson's disease. Cellular and molecular life sciences: CMLS. 2012;69(7):1153–65. Epub 2011/10/11. 10.1007/s00018-011-0850-z . 21984601 PMC11114858

[33] Sczelecki S, Besse-Patin A, Abboud A, Kleiner S, Laznik-Bogoslavski D, Wrann CD, et al Loss of Pgc-1α expression in aging mouse muscle potentiates glucose intolerance and systemic inflammation. American journal of physiology Endocrinology and metabolism. 2014;306(2):E157–67. Epub 2013/11/28. 10.1152/ajpendo.00578.2013 . 24280126 PMC4073996

[34] Nijland PG, Witte ME, van Het Hof B, van der Pol S, Bauer J, Lassmann H, et al Astroglial PGC-1α increases mitochondrial antioxidant capacity and suppresses inflammation: implications for multiple sclerosis. Acta neuropathologica communications. 2014;2(1):170 10.1186/s40478-014-0170-2 25492529 PMC4268800

[35] Bjorkqvist M, Wild EJ, Tabrizi SJ. Harnessing immune alterations in neurodegenerative diseases. Neuron. 2009;64(1):21–4. 10.1016/j.neuron.2009.09.034 . 19840543

[36] Xie W, Barr CL, Kim A, Yue F, Lee AY, Eubanks J, et al Base-resolution analyses of sequence and parent-of-origin dependent DNA methylation in the mouse genome. Cell. 2012;148(4):816–31. Epub 2012/02/22. 10.1016/j.cell.2011.12.035 22341451 PMC3343639

[37] Lister R, Mukamel EA, Nery JR, Urich M, Puddifoot CA, Johnson ND, et al Global epigenomic reconfiguration during mammalian brain development. Science. 2013;341(6146):1237905 Epub 2013/07/06. 10.1126/science.1237905 23828890 PMC3785061

[38] Guo JU, Su Y, Shin JH, Shin J, Li H, Xie B, et al Distribution, recognition and regulation of non-CpG methylation in the adult mammalian brain. Nat Neurosci. 2014;17(2):215–22. Epub 2013/12/24. 10.1038/nn.3607 . 24362762 PMC3970219

[39] Varley KE, Gertz J, Bowling KM, Parker SL, Reddy TE, Pauli-Behn F, et al Dynamic DNA methylation across diverse human cell lines and tissues. Genome Res. 2013;23(3):555–67. Epub 2013/01/18. 10.1101/gr.147942.112 23325432 PMC3589544

[40] Walsh CP, Bestor TH. Cytosine methylation and mammalian development. Genes Dev. 1999;13(1):26–34. Epub 1999/01/14. 9887097 10.1101/gad.13.1.26 PMC316374

[41] Baylin SB. DNA methylation and gene silencing in cancer. Nat Clin Pract Oncol. 2005;2 Suppl 1:S4–11. Epub 2005/12/13. 10.1038/ncponc0354 . 16341240

[42] Hermann A, Goyal R, Jeltsch A. The Dnmt1 DNA-(cytosine-C5)-methyltransferase methylates DNA processively with high preference for hemimethylated target sites. J Biol Chem. 2004;279(46):48350–9. Epub 2004/09/02. 10.1074/jbc.M403427200 . 15339928

[43] Feng J, Zhou Y, Campbell SL, Le T, Li E, Sweatt JD, et al Dnmt1 and Dnmt3a maintain DNA methylation and regulate synaptic function in adult forebrain neurons. Nat Neurosci. 2010;13(4):423–30. Epub 2010/03/17. 10.1038/nn.2514 20228804 PMC3060772

[44] Feng J, Chang H, Li E, Fan G. Dynamic expression of de novo DNA methyltransferases Dnmt3a and Dnmt3b in the central nervous system. J Neurosci Res. 2005;79(6):734–46. Epub 2005/01/27. 10.1002/jnr.20404 . 15672446

[45] Chestnut BA, Chang Q, Price A, Lesuisse C, Wong M, Martin LJ. Epigenetic regulation of motor neuron cell death through DNA methylation. J Neurosci. 2011;31(46):16619–36. Epub 2011/11/18. 10.1523/JNEUROSCI.1639-11.2011 22090490 PMC3238138

[46] Lin J, Wu H, Tarr PT, Zhang CY, Wu Z, Boss O, et al Transcriptional co-activator PGC-1 α drives the formation of slow-twitch muscle fibres. Nature. 2002;418(6899):797–801. Epub 2002/08/16. 10.1038/nature00904 . 12181572

[47] Wenz T, Diaz F, Spiegelman BM, Moraes CT. Activation of the PPAR/PGC-1α pathway prevents a bioenergetic deficit and effectively improves a mitochondrial myopathy phenotype. Cell metabolism. 2008;8(3):249–56. Epub 2008/09/03. 10.1016/j.cmet.2008.07.006 18762025 PMC2613643

[48] Li L, Pan R, Li R, Niemann B, Aurich AC, Chen Y, et al Mitochondrial biogenesis and peroxisome proliferator-activated receptor-gamma coactivator-1α (PGC-1α) deacetylation by physical activity: intact adipocytokine signaling is required. Diabetes. 2011;60(1):157–67. 10.2337/db10-0331 20929977 PMC3012167

[49] Wu Z, Puigserver P, Andersson U, Zhang C, Adelmant G, Mootha V, et al Mechanisms controlling mitochondrial biogenesis and respiration through the thermogenic coactivator PGC-1. Cell. 1999;98(1):115–24. Epub 1999/07/21. 10.1016/S0092-8674(00)80611-X . 10412986

[50] Eisele PS, Salatino S, Sobek J, Hottiger MO, Handschin C. The peroxisome proliferator-activated receptor gamma coactivator 1α/beta (PGC-1) coactivators repress the transcriptional activity of NF-kappaB in skeletal muscle cells. J Biol Chem. 2013;288(4):2246–60. Epub 2012/12/12. 10.1074/jbc.M112.375253 23223635 PMC3554897

[51] Valle I, Alvarez-Barrientos A, Arza E, Lamas S, Monsalve M. PGC-1α regulates the mitochondrial antioxidant defense system in vascular endothelial cells. Cardiovasc Res. 2005;66(3):562–73. Epub 2005/05/26. 10.1016/j.cardiores.2005.01.026 . 15914121

[52] Shin JH, Ko HS, Kang H, Lee Y, Lee YI, Pletinkova O, et al PARIS (ZNF746) repression of PGC-1α contributes to neurodegeneration in Parkinson's disease. Cell. 2011;144(5):689–702. 10.1016/j.cell.2011.02.010 21376232 PMC3063894

[53] Canto C, Auwerx J. PGC-1α, SIRT1 and AMPK, an energy sensing network that controls energy expenditure. Curr Opin Lipidol. 2009;20(2):98–105. Epub 2009/03/12. 10.1097/MOL.0b013e328328d0a4 19276888 PMC3627054

[54] Wan Z, Root-McCaig J, Castellani L, Kemp BE, Steinberg GR, Wright DC. Evidence for the role of AMPK in regulating PGC-1 α expression and mitochondrial proteins in mouse epididymal adipose tissue. Obesity (Silver Spring). 2014;22(3):730–8. Epub 2013/08/22. 10.1002/oby.20605 . 23963743

[55] Dauer W, Przedborski S. Parkinson's disease: mechanisms and models. Neuron. 2003;39(6):889–909. . 12971891 10.1016/s0896-6273(03)00568-3

[56] Hsiao YH, Lin CI, Liao H, Chen YH, Lin SH. Palmitic acid-induced neuron cell cycle G2/M arrest and endoplasmic reticular stress through protein palmitoylation in SH-SY5Y human neuroblastoma cells. International journal of molecular sciences. 2014;15(11):20876–99. 10.3390/ijms151120876 25402647 PMC4264201

[57] Baldwin AC, Green CD, Olson LK, Moxley MA, Corbett JA. A role for aberrant protein palmitoylation in FFA-induced ER stress and beta-cell death. American journal of physiology Endocrinology and metabolism. 2012;302(11):E1390–8. 10.1152/ajpendo.00519.2011 22436701 PMC3378068

[58] Bartoszewski R, Rab A, Twitty G, Stevenson L, Fortenberry J, Piotrowski A, et al The mechanism of cystic fibrosis transmembrane conductance regulator transcriptional repression during the unfolded protein response. J Biol Chem. 2008;283(18):12154–65. 10.1074/jbc.M707610200 . 18319256

[59] Deng J, Lu PD, Zhang Y, Scheuner D, Kaufman RJ, Sonenberg N, et al Translational repression mediates activation of nuclear factor kappa B by phosphorylated translation initiation factor 2. Mol Cell Biol. 2004;24(23):10161–8. 10.1128/MCB.24.23.10161-10168.2004 15542827 PMC529034

[60] Hu P, Han Z, Couvillon AD, Kaufman RJ, Exton JH. Autocrine tumor necrosis factor α links endoplasmic reticulum stress to the membrane death receptor pathway through IRE1α-mediated NF-kappaB activation and down-regulation of TRAF2 expression. Mol Cell Biol. 2006;26(8):3071–84. 10.1128/MCB.26.8.3071-3084.2006 16581782 PMC1446932

[61] Barres R, Osler ME, Yan J, Rune A, Fritz T, Caidahl K, et al Non-CpG methylation of the PGC-1α promoter through DNMT3B controls mitochondrial density. Cell metabolism. 2009;10(3):189–98. Epub 2009/09/03. 10.1016/j.cmet.2009.07.011 . 19723495

