# Supplementary figures and images for: PGC−1α Promoter Methylation in Parkinson’s Disease
Source: PLoS One. 2015 Aug 28;10(8):e0134087. doi: 10.1371/journal.pone.0134087 (PMC4552803; doi:10.1371/journal.pone.0134087)

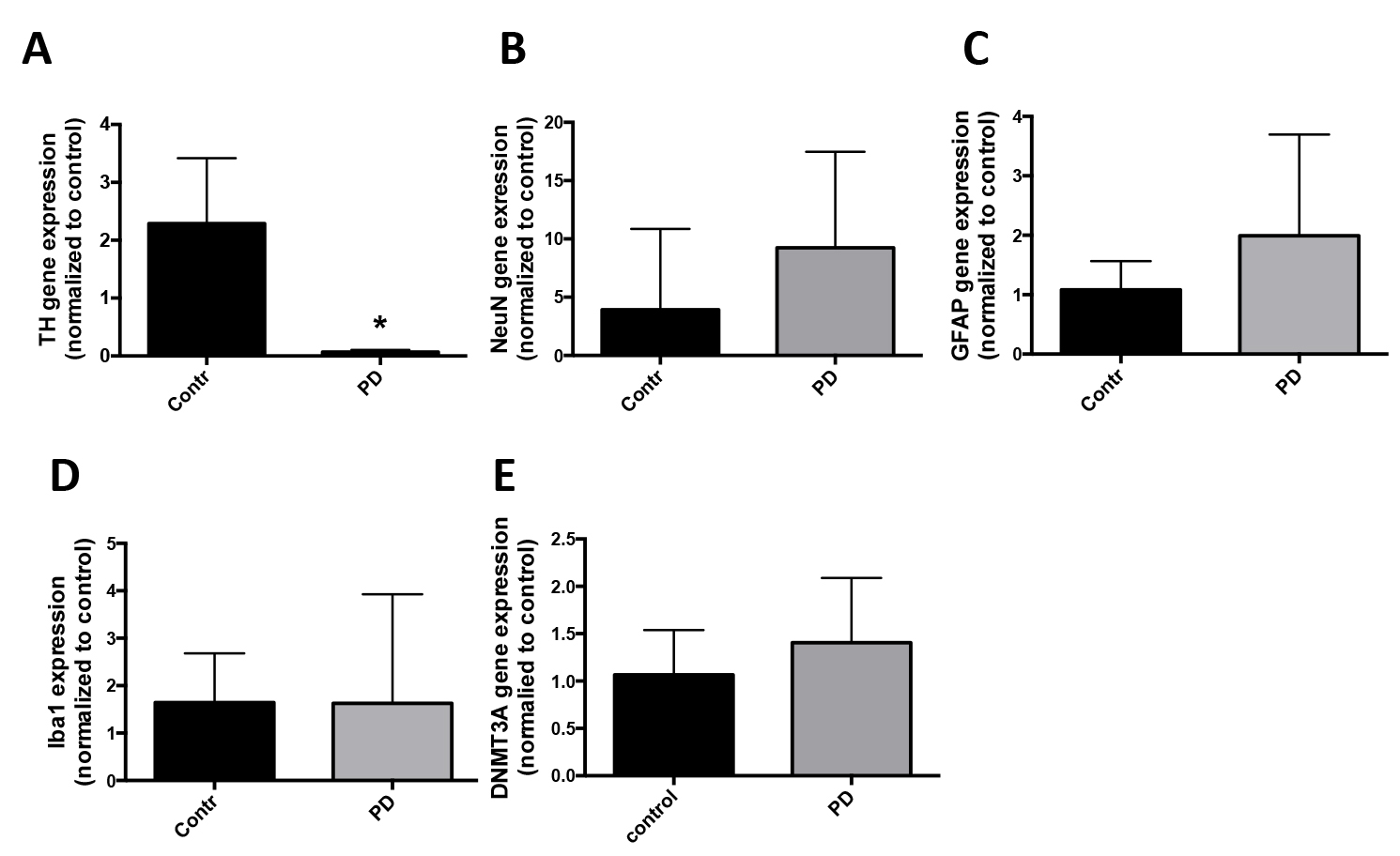

Supplement: S1 Fig — CNS cell markers TH (A), NeuN (B), GFAP (C) and Iba1 (D) gene expression was determined by real time PCR. E. DNMT3A gene expression was measured by real time PCR. Results were presented as mean±SEM (*p<0.05). Differences between groups were determined by unpaired Student’s t test. (TIF) [file pone.0134087.s001.tif]

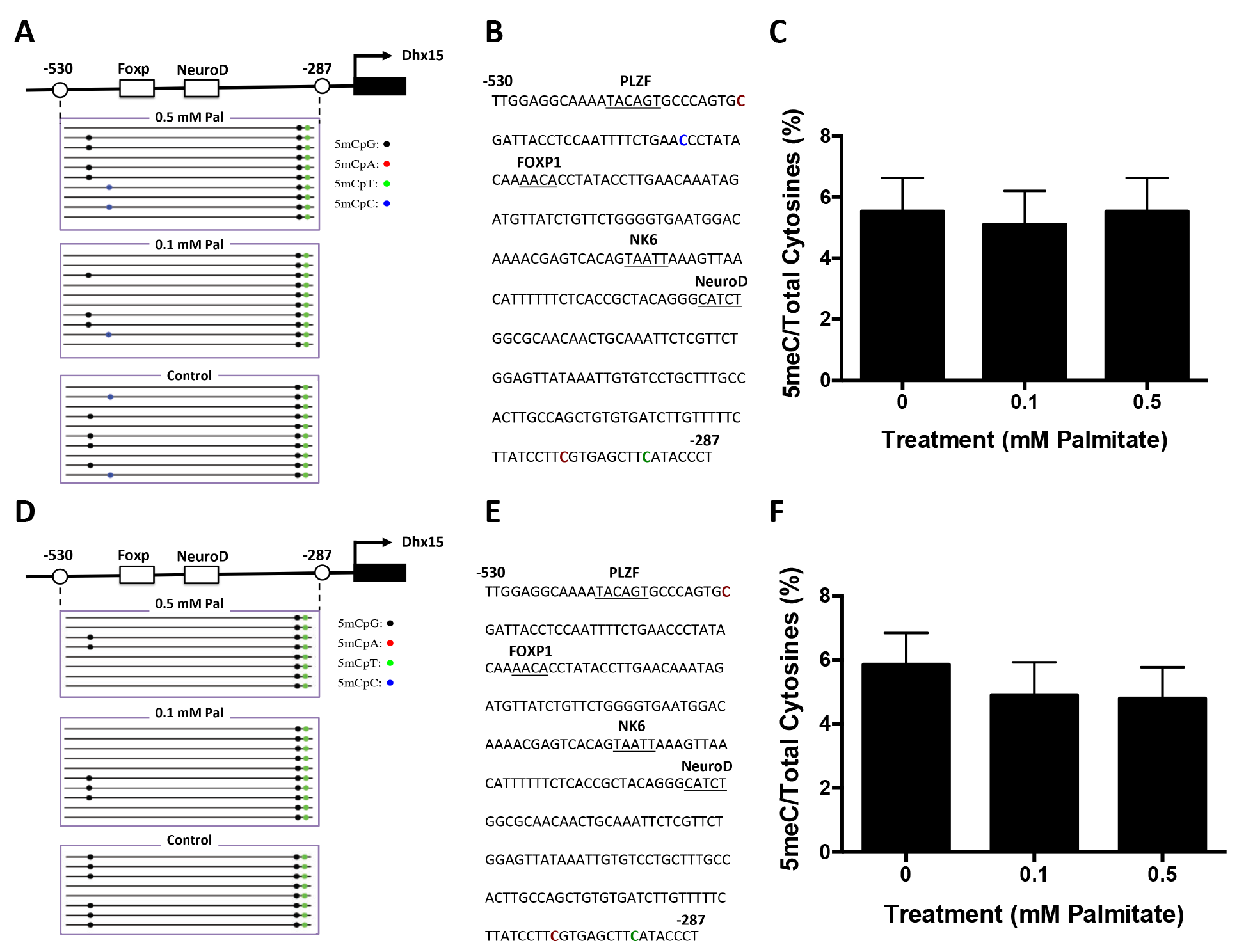

Supplement: S2 Fig — Primary microglia and astrocytes were treated with 0.1 and 0.5 mM Palmitate for 48 hours. Genomic DNA was isolated for bisulfite sequencing. Visualization of the bisulfite sequencing results for DHX15 promoter was completed using MethTools 3.0. A. Graphical depiction of the DHX15 promoter region (-530 to -287) and location of methylated ctyosines in primary microglia. B. Methylation sequencing region in the DHX15 promoter in primary microglia. Important transcription factors are underlined. Methylated CpGs are highlighted in brown, CpAs in red, CpTs in green and CpCs in blue. C. Quantitation of cytosine methylation levels of DHX15 promoter in primary microglia. D. Graphical depiction of the DHX15 promoter region (-530 to -287) and location of methylated ctyosines in primary astrocytes. E. Methylation region sequenced in the DHX15 promoter. Important transcription factors are underlined. Methylated CpGs are highlighted in brown, CpAs in red, CpTs in green and CpCs in blue. F. Quantitation of cytosine methylation levels of DHX15 promoter in primary astrocytes. Results were presented as mean±SEM, ANOVA with Student-Newman-Keuls post hoc analysis (C and F). (TIF) [file pone.0134087.s002.tif]
